# ContiTE: continuous manifold MoE for few-shot cross-tissue mRNA translation efficiency prediction

**DOI:** 10.1093/bib/bbag518

**Published:** 2026-09-21

**Authors:** Yuxiao Wei, Qi Zhang, Sainan Huo, Xuezhong Zhou

**Affiliations:** School of Computer Science and Technology, Beijing Jiaotong University, Beijing 100044, China; School of Computer Science and Engineering, Nanjing University of Science and Technology, 200 Xiaolingwei, Nanjing 210094, China; School of Computer Science and Technology, Beijing Jiaotong University, Beijing 100044, China; School of Computer Science and Technology, Beijing Jiaotong University, Beijing 100044, China

**Keywords:** mRNA translation efficiency, continuous manifold, mixture of experts, few-shot adaptation, test-time adaptation

## Abstract

Predicting mRNA translation efficiency across diverse tissues is a critical yet challenging task due to the phenomenon of concept shift, where identical genomic sequences exhibit distinct functional profiles across varying cellular environments. Existing static models are often limited by fixed parameterization, struggling to capture these continuous regulatory variations without requiring computationally expensive fine-tuning. To address these limitations, we propose ContiTE, a continuous manifold mixture-of-experts (MoE) framework explicitly designed for efficient few-shot cross-tissue adaptation. Unlike traditional MoE architectures that rely on discrete output-space mixing, ContiTE operates on a continuous parameter manifold by dynamically synthesizing domain-specific weights through a context-aware hyper-router that linearly combines shared atomic basis kernels. This paradigm enables smooth interpolation of translational rules and provides a flexible mechanism to model complex, context-dependent biological regulation. Furthermore, we introduce a gradient-based test-time adaptation strategy that allows the model to rapidly align to new, unseen tissues by solely optimizing low-dimensional context embeddings while keeping the backbone parameters frozen. Experimental results on a comprehensive human and mouse atlas demonstrate that ContiTE significantly outperforms state-of-the-art methods in few-shot scenarios, improving average Pearson correlation coefficient by 12.09% and $R^{2}$ by 18.22%. By mitigating negative transfer and requiring only limited target-domain data for calibration, ContiTE provides a computational framework for tissue-specific mRNA translation-efficiency prediction and demonstrates a parameter-reconstruction strategy that may be extensible to other cross-domain sequence-learning tasks, subject to task-specific validation.

## Introduction

mRNA translation serves as the pivotal rate-limiting step that converts genetic information into functional proteins, with its efficiency directly governing cellular proteome abundance [1, 2]. With the advent of mRNA vaccines and protein replacement therapies, precise regulation of mRNA translation efficiency (TE) within specific tissues has emerged as a core challenge in synthetic biology [3, 4]. However, the translation process is orchestrated by the synergistic interplay between sequence features (cis-elements) and intracellular environmental factors (trans-factors, e.g. tRNA abundance, RBP concentration), which critically modulate tissue-specific translation dynamics [5], leading to the phenomenon of “one sequence, multiple fates” [6]. From a machine learning perspective, this constitutes a prototypical Concept Shift: while the input distribution *P(X)* remains constant, the conditional probability distribution *P(Y|X)* changes drastically across different domains (tissues) [7].

Computational modeling of mRNA translation regulation has undergone a significant paradigm shift from manual feature engineering to end-to-end deep representation learning. Early research primarily focused on mining individual sequence features, employing linear regression or boosted tree models to integrate physicochemical properties such as sequence length, codon bias, Kozak sequence strength, and secondary structure folding energy to predict TE [8–10]. With breakthroughs in massively parallel reporter assays, the focus shifted toward utilizing convolutional neural networks (CNNs) to decipher regulatory grammars in specific regions. Representative works such as Optimus 5-prime [11] and Frame Pool [12] successfully established mappings between 5$^\prime $ untranslated region (5$^\prime $ UTR) sequences and ribosome load in specific cell lines using massive synthetic reporter data. To further capture the synergy of full-length sequences, recent studies have begun constructing deep learning architectures for endogenous mRNA; for instance, Saluki [13] attempts to capture long-range dependencies by combining CNNs with recurrent neural networks. The latest advancement is epitomized by RiboNN [14], which constructed a transcriptome-wide atlas encompassing human and mouse species across multiple cell types. Adopting a multi-task deep CNN architecture, RiboNN not only achieved end-to-end prediction from full-length mRNA sequences to TE but also revealed the evolutionary conservation of translational regulatory rules across mammals via cross-species analysis, successfully extending model capabilities to therapeutic mRNA design through transfer learning. Concurrently, integrated deep learning frameworks have been successfully deployed to optimize mRNA sequences for enhanced expression [15], while multi-task learning paradigms have expanded our capacity to decode the functional landscapes of diverse RNA species [16]. Furthermore, inspired by the success of natural language processing, Transformer-based architectures and large-scale pre-trained foundation models have recently been introduced to genomics. For example, RNAdegformer [17] leverages self-attention mechanisms to capture long-range sequence dependencies, while mRNABERT [18] and other advanced foundation models [19] utilize massive unannotated transcriptomic data to learn universal representations. Concurrently, deep architectures have been extended to map complex RNA modification and expression dynamics across diverse biological settings [20].

Despite these advancements in single domains, existing research faces three fundamental mismatches regarding problem definition and generalization mechanisms when addressing complex cross-tissue TE prediction. First, existing transfer learning work in bioinformatics primarily focuses on cross-species adaptation, which essentially addresses Covariate Shift (input distribution shift), often using adversarial training or feature alignment to bridge sequence discrepancies [21–24]. However, this study confronts the more intractable pure Concept Shift, where traditional domain alignment fails to model mechanisms where identical sequences are assigned different translation rates in different environments [7], leaving models helpless against environment-dependent regulation. This highlights an urgent need for robust domain adaptation methodologies to disentangle environmental heterogeneity in complex transcriptomic landscapes [25]. Existing solutions typically rely on multi-task learning or fine-tuning; while effective, the high computational cost and massive data requirements of fine-tuning make them ill-suited for few-shot adaptation to new tissues [26].

Second, biological state transitions (e.g. cell differentiation trajectories) are inherently continuous processes lying on a low-dimensional manifold [27, 28]. More recently, Zhang *et al.* [29] developed a health state manifold based on multi-omics data to quantify individual health states across disease progression and recovery, providing complementary support for manifold-based representations of continuously varying biological states. To enhance environmental adaptability, mixture-of-experts (MoE) models have recently shown potential in genomics. For instance, models like SPACE [30] have proposed cross-species synergistic expert architectures, using sparse routing to balance species-specific and conserved features. However, standard MoE models typically employ a Top-K “hard selection” mechanism of discrete experts in the output space [31]. This discreteness precludes smooth interpolation between experts and fragments continuous biological states, making it difficult to simulate subtle, gradual regulatory logic, while independent experts inevitably lead to linear parameter growth.

Finally, regarding representation learning mechanisms, the existing paradigm is constrained by the bottleneck of “static parameterization.” Traditional models strive to learn a fixed set of network weights to extract universal sequence features across all environments. However, real-world biological regulation is highly context-dependent; forcing a static model with a fixed feature extractor to adapt to all environments leads to severe inter-task interference and negative transfer [32]. This “ossification of feature space” deprives models of the ability to dynamically reconfigure at the parameter level, failing to mimic how biological systems flexibly adjust underlying perception rules based on environmental signals [33].

**Figure 1:**
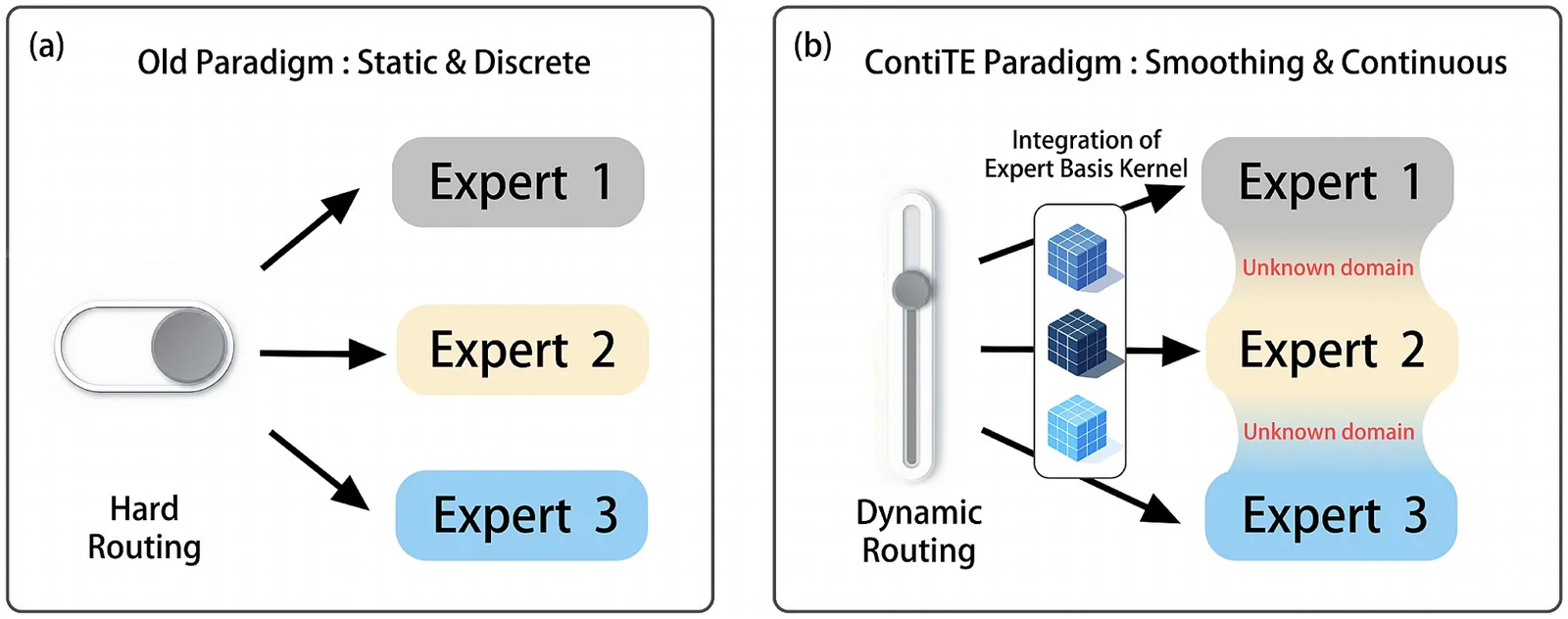
Overcoming rigid modeling with ContiTE. (a) Challenges: The inflexibility of static parameters and discrete routing for concept shift. (b) Solution: Continuous expert synthesis in weight space via “Atomic Bases.”

To address these challenges, we propose ContiTE, a sequence modeling framework designed for few-shot cross-tissue mRNA translation-efficiency prediction under biological concept shift (Fig. 1). Our core insight is that although translational regulatory rules vary across tissues, they are not mutually independent but lie on a latent low-dimensional parameter manifold. Therefore, we propose a novel Weight-Space MoE paradigm of “Atomization and Reconstruction”: we decompose expert capabilities into a set of shared atomic basis kernels and project their combinations onto a continuous parameter manifold, continuously reconfiguring these atoms in the weight space via a hypernetwork. This design enables ContiTE to perform smooth interpolation in parameter space and synthesize context-dependent experts for different tissue environments, thereby providing a flexible mechanism for adaptation under cross-tissue concept shift.

The main contributions of this paper are summarized as follows:

- **Continuous manifold MoE based on atomic bases.** We redefine the morphology of “experts” in MoE. Unlike traditional MoE relying on discrete sub-networks, we decouple expert capabilities into a set of learnable and shared atomic basis kernels. By sinking the mixing mechanism into the parameter space, ContiTE can continuously “reconstruct” dynamic experts for specific environments via linear combinations of these bases, enabling precise modeling of continuously changing biophysical manifolds.
- **Parameter-efficient test-time adaptation (TTA).** We propose an efficient transfer strategy that requires no retraining of the backbone network. Leveraging ContiTE’s expert reconstruction property, with only a few samples from the target domain, the model can rapidly calibrate a low-dimensional context embedding vector via gradient descent. This allows the atomic bases to reconstruct dynamic experts adapted to unseen new tissues within seconds, significantly enhancing cross-domain generalization.
- **State-of-the-art performance.** ContiTE achieves state-of-the-art performance ($R^{2}=0.671$) in few-shot, tissue-specific mRNA TE prediction, significantly outperforming existing methods, including large-scale pre-trained models.
- **Interpretability via visualization.** We conduct multi-dimensional visualization analyses to decode ContiTE’s decision logic, going beyond traditional “black box” approaches. By revealing the biological clustering properties of tissue representations, precise focusing on key regulatory elements, and efficient cross-domain adaptation mechanisms, we provide an empirical basis for the interpretability of the model’s regulatory decisions.

## Materials and methods

### Datasets and experimental setup

To comprehensively evaluate the cross-tissue generalization performance of ContiTE, we used a transcriptome-wide benchmark comprising 11 798 human and 11 575 mouse genes derived from the processed atlas released with RiboNN [14]. This dataset spans the full 5$^\prime $ UTR, CDS, and 3$^\prime $ UTR regions and covers 96 human and 46 mouse cell or tissue contexts. Importantly, the human and mouse atlases were treated as two separate species-specific benchmarks. Within each species, tissues were partitioned into source, validation, and target domains, and all adaptation and evaluation were performed between tissues of the same species. No direct human-to-mouse or mouse-to-human transfer was conducted; therefore, the principal task evaluated in this study is few-shot cross-tissue, rather than cross-species, TE prediction. The evaluated atlas includes pluripotent stem cells (e.g. H1-hESC and mESC), differentiated tissues and cell states [e.g. Bone, dorsal root ganglion (DRG), Kidney, and ES-derived neurons], immortalized cell lines (e.g. HEK293T and HeLa), and cancer-associated samples or cell lines [e.g. hepatocellular carcinoma (HCC) tumor, B16 melanoma, A549, and MDA-MB-231]. Thus, the benchmark is not restricted to normal differentiated tissues, although it does not encompass controlled stress conditions, rare clinical biopsies, or all forms of pathological translational reprogramming. Detailed information on the human and mouse subsets of the RiboNN atlas, including the original source files and direct download links, is provided in Supplementary Section Dataset Sources and Accessibility and Supplementary Table S1.

For ground-truth labeling, we integrated high-depth Ribosome Profiling (Ribo-seq) and RNA-seq data to calculate the log-transformed TE (log-TE), effectively decoupling translation kinetics from transcriptional background noise. During preprocessing, genes with low expression (RPKM*<1*) or anomalous lengths were rigorously filtered. Crucially, we adopted a Tissue-specific Split strategy—reserving distinct tissues as test sets—to strictly assess the model’s few-shot generalization capabilities across unseen biological contexts.

### Model architecture

Formally, given an mRNA sequence *x* and a specific tissue context domain *d*, the goal of TE prediction is to learn a mapping function *f(x, d) → y* to estimate the protein synthesis rate *y* of the sequence within that tissue. To scale model capacity for varying contexts, standard MoE utilizes a gating network *G(· )* to compute sparse routing weights and aggregates the outputs of distinct expert networks $\{E_{k}\}_{k=1}^{K}$ via weighted summation:


(1) \begin{align*}& y = \sum_{k=1}^{K} G(x)_{k} \cdot E_{k}(x)\end{align*}

However, this paradigm, referred to as “output-space mixing,” relies on discrete gating where different experts process the input independently. It struggles to capture the continuous regulatory manifolds inherent in biology. Furthermore, when confronting unseen tissue domains, routing strategies dependent on instance inputs often fail due to concept shift.

**Figure 2:**
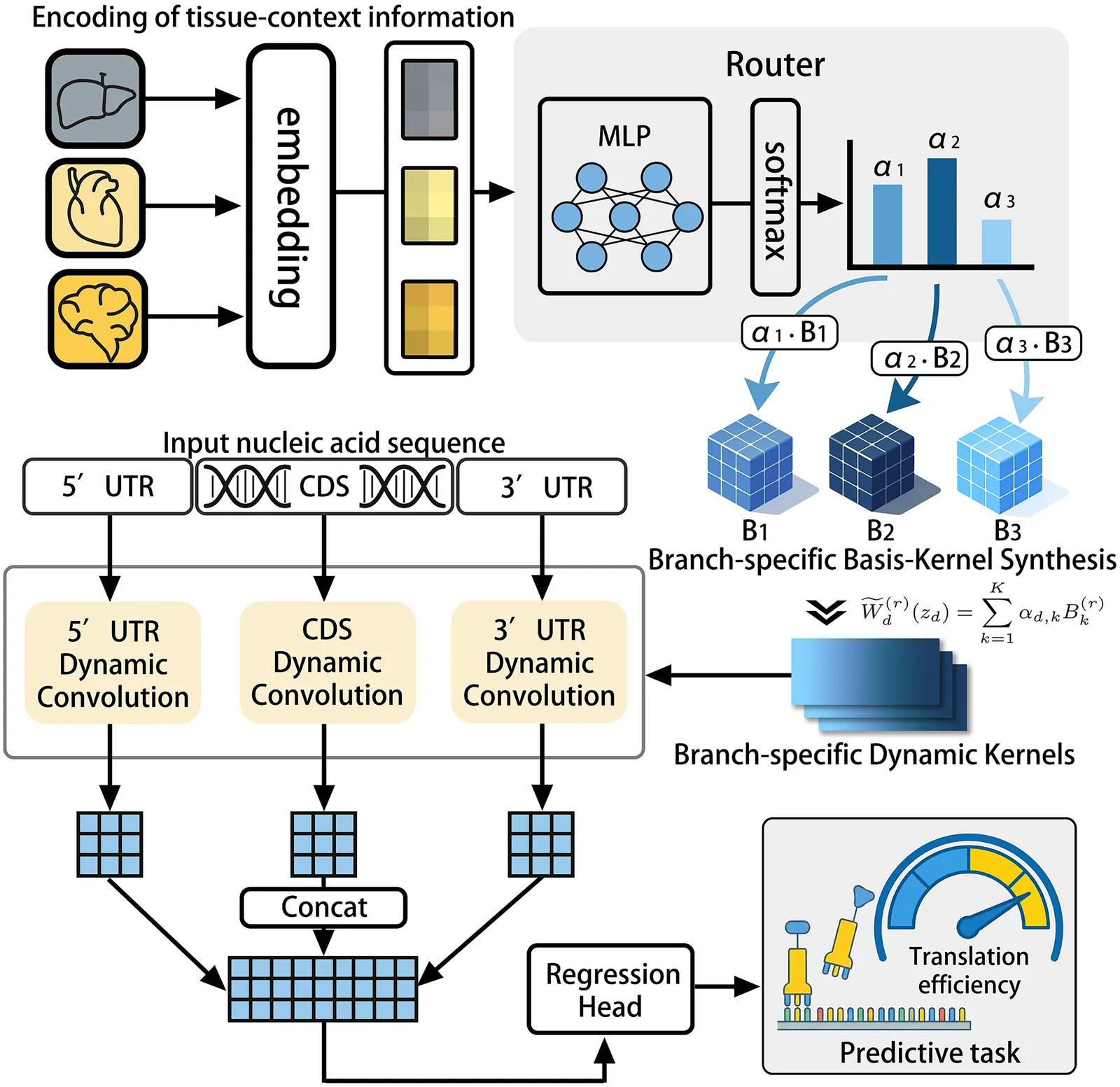
The proposed ContiTE architecture. The Context Hyper-Router maps a tissue context embedding to tissue-conditioned mixing coefficients. The 5' UTR, CDS, and 3' UTR branches maintain separately parameterized atomic basis-kernel banks because they use different convolutional configurations. The same tissue-conditioned mixing coefficients synthesize one dynamic convolutional kernel for each branch. Following branch-specific dynamic convolution, nonlinear activation, and global max pooling, the three branch representations are concatenated and passed to the regression MLP to produce the scalar translation-efficiency prediction.

**Figure 3:**
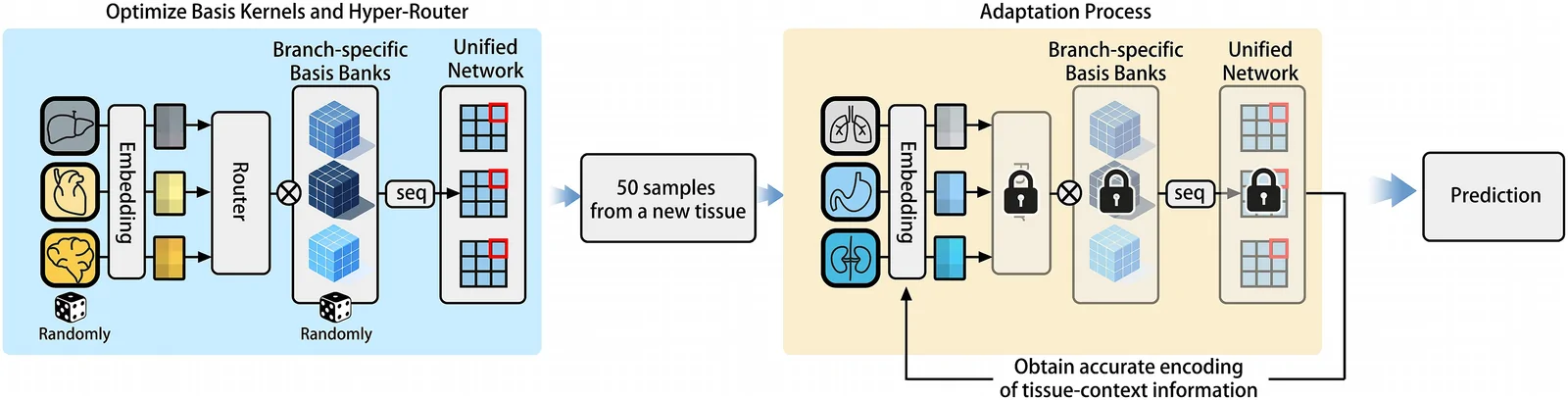
The learning and adaptation workflow. The schematic illustrates the Meta-Training phase where the router and encoder are jointly optimized on source domains to learn shared regulatory manifolds, followed by the TTA phase where only the tissue-specific context embeddings are updated via gradient descent to generalize to unseen target domains.

To address these challenges, we propose ContiTE, a framework designed for few-shot adaptation based on a Continuous Manifold MoE architecture. The framework comprises two synergistic components: a Structure-Aware Sequence Encoder and a Context Hyper-Router, parameterized by $\theta _{enc}$ and $\theta _{router}$, respectively. These components collaborate to achieve dynamic inference:

1. The Hyper-Router first maps a learnable tissue context embedding $z_{d}$ to a tissue-conditioned mixing coefficient vector $\alpha _{d}$. Because the 5' UTR, CDS, and 3' UTR branches use different convolutional configurations, each branch maintains a separately parameterized atomic basis-kernel bank. The same tissue-conditioned coefficient vector is applied to the three branch-specific basis banks to synthesize one dynamic convolutional kernel for each branch.
2. The Structure-Aware Sequence Encoder applies the synthesized kernels to the corresponding sequence regions. The resulting branch-level representations are aggregated and concatenated, and the concatenated representation is passed to the regression Multilayer Perceptron (MLP) to produce the scalar prediction $\hat{y}$.
  Crucially, ContiTE explores gradient-based TTA techniques. By optimizing solely the context embedding $z_{d}$ while keeping other parameters frozen during the inference phase, the model can resolve concept shift and enhance generalization capabilities on unseen tissues. The detailed model architecture is illustrated in Fig. 2, and the workflows for meta-training and TTA are depicted in Fig. 3. We further elaborate on these components in the following three subsections.

### Context-driven weight synthesis

The core innovation of ContiTE lies in shifting the paradigm from discrete expert selection to continuous parameter reconstruction on a latent manifold. Because the 5$^\prime $ UTR, CDS, and 3$^\prime $ UTR branches employ different convolutional configurations, we maintain a separate atomic basis-kernel bank for each branch:


(2) \begin{align*}& \begin{aligned} B^{(r)} &= \left\{ B^{(r)}_{1}, B^{(r)}_{2}, \ldots, B^{(r)}_{K} \right\}\kern-2pt, \qquad r \in \mathcal{R}, \\ \mathcal{R} &= \left\{ 5^{\prime}\mathrm{UTR}, \mathrm{CDS}, 3^{\prime}\mathrm{UTR} \right\}\kern-2pt. \end{aligned}\end{align*}

Here, $\mathcal{R}$ denotes the set of three functional sequence branches; *r* indexes a specific branch; $\mathcal{B}^{(r)}$ is the atomic basis-kernel bank of branch *r*; *K* is the number of atomic basis kernels in each bank; and $B_{k}^{(r)}$ denotes the *k*th basis kernel of branch *r*. The basis kernels within each branch-specific bank are shared across tissue domains but are parameterized independently from the basis kernels in the other branches.

For tissue domain *d*, the Context Hyper-Router maps the learnable context embedding $z_{d}\in \mathbb{R}^{D_{z}}$ to a coefficient vector on the probability simplex:


(3) \begin{align*}& \boldsymbol{\alpha}_{d} = \operatorname{Softmax} \left( \operatorname{MLP}(z_{d}) \right) \in \Delta^{K-1}.\end{align*}

Here, *d* indexes the tissue domain; $z_{d}$ is its $D_{z}$-dimensional context embedding; $\boldsymbol{\alpha }_{d} = [\alpha _{d,1},\ldots ,\alpha _{d,K}]^\top $ is the tissue-conditioned mixing coefficient vector; and $\Delta ^{K-1}$ denotes the standard *(K-1)*-dimensional probability simplex, such that $\alpha _{d,k}\geq 0$ and $\sum _{k=1}^{K}\alpha _{d,k}=1$.

The same tissue-conditioned coefficient vector is applied to the corresponding basis bank of each sequence branch to synthesize its dynamic convolutional kernel:


(4) \begin{align*}& \widetilde{W}^{(r)}_{d}(z_{d}) = \sum_{k=1}^{K} \alpha_{d,k}B^{(r)}_{k}, \qquad r\in\mathcal{R}.\end{align*}

Here, $\widetilde{W}^{(r)}_{d}(z_{d})$ denotes the dynamic convolutional kernel synthesized for tissue *d* and sequence branch *r*, while $\alpha _{d,k}$ specifies the contribution of the *k*th atomic basis kernel $B_{k}^{(r)}$.

Importantly, these branch-specific atomic basis kernels are latent computational components learned end-to-end in parameter space. They are not predefined to maintain a one-to-one correspondence with individual trans-regulatory factors, such as tRNA abundance, RNA-binding protein activity, mRNA structural stability, or translation initiation factor abundance. Accordingly, changes in the mixing coefficients or synthesized branch-specific weights should be interpreted as aggregate adaptations to tissue-specific regulatory contexts rather than as direct quantitative measurements of particular molecular factors.

For target domains whose regulatory patterns can be represented by the learned basis span, adjusting $z_{d}$ enables interpolation within the convex hull of the basis kernels. The mechanism is therefore designed primarily to mitigate continuous forms of concept shift and does not guarantee accurate extrapolation to qualitatively novel regulatory mechanisms outside the source-domain manifold.

### Structure-aware dynamic architecture

The embedded mRNA sequence is divided into three functional regions: the 5$^\prime $ UTR, CDS, and 3$^\prime $ UTR. Each region is processed by an independently parameterized dynamic-convolution branch. For branch $r\in \mathcal{R}$, the multi-channel one-dimensional convolution is defined as


(5) \begin{align*}& \begin{aligned} U^{(r)}_{d,o,t} &= b^{(r)}_{o} + \sum_{\substack{ 1\leq c\leq C_{\mathrm{in}}\\ 0\leq j<S_{r} }} \widetilde{W}^{(r)}_{d,o,c,j} X^{(r)}_{c,t s_{r}+j}, \\ H^{(r)}_{d,o,t} &= \sigma \left( U^{(r)}_{d,o,t} \right)\kern-2pt. \end{aligned}\end{align*}

Here, $X^{(r)}_{c,\ell }$ denotes the embedded input feature at sequence position *ℓ * and input channel *c* in branch *r*; $\widetilde{W}^{(r)}_{d,o,c,j}$ is the synthesized kernel coefficient connecting input channel *c* to output channel *o* at kernel position *j*; $U^{(r)}_{d,o,t}$ and $H^{(r)}_{d,o,t}$ denote the pre-activation and post-activation features, respectively, at output position *t*; $b_{o}^{(r)}$ is the bias associated with output channel *o*; $C_{\mathrm{in}}=16$ and $C_{\mathrm{out}}=32$ are the numbers of input and output channels; $S_{r}$ and $s_{r}$ are the branch-specific kernel size and stride; and $\sigma (\cdot )$ denotes the nonlinear activation function. The index *t* ranges over all valid output positions of branch *r*.

Motivated by the triplet organization of coding sequences and codon-wise ribosomal decoding, the CDS branch uses a kernel size of 3 and a stride of 3. This configuration processes non-overlapping nucleotide triplets aligned from the beginning of the preprocessed CDS and therefore introduces a codon-periodicity-oriented architectural inductive bias. It should not be interpreted as a general signal-processing theorem or as guaranteeing phase invariance or noise suppression. Its practical contribution is evaluated empirically through the w/o Codon ablation, in which the stride-3 CDS convolution is replaced by a standard stride-1 convolution.

Following dynamic convolution and nonlinear activation, global max pooling is applied over the sequence-length dimension of each branch:


(6) \begin{align*}& q^{(r)}_{d,o} = \max_{1\leq t\leq T_{r}} H^{(r)}_{d,o,t}.\end{align*}

Here, $T_{r}$ is the number of output positions in branch *r*, and $q^{(r)}_{d,o}$ is the pooled feature of output channel *o*. The resulting branch representation is $q_{d}^{(r)}\in \mathbb{R}^{C_{\mathrm{out}}}$.

The three branch-level representations are concatenated as


(7) \begin{align*}& q_{d} = q_{d}^{(5^\prime\mathrm{UTR})} \mathbin{\Vert} q_{d}^{(\mathrm{CDS})} \mathbin{\Vert} q_{d}^{(3^\prime\mathrm{UTR})} \in \mathbb{R}^{3C_{\mathrm{out}}} = \mathbb{R}^{96}.\end{align*}

Here, *‖ * denotes vector concatenation, and $q_{d}$ is the unified 96-dimensional sequence representation for tissue domain *d*.

The scalar translation-efficiency prediction is then produced by the regression MLP:


(8) \begin{align*}& \widehat{y} = \operatorname{MLP}_{\mathrm{reg}}(q_{d}).\end{align*}

Here, $\operatorname{MLP}_{\mathrm{reg}}(\cdot )$ denotes the regression head and $\widehat{y}\in \mathbb{R}$ denotes the predicted log-transformed translation-efficiency value.

### Gradient-based test-time adaptation

When deployed to an unseen target domain, traditional static models typically exhibit performance collapse due to the lack of a corresponding context embedding $z_{target}$. ContiTE leverages its parameter generation property to propose an efficient TTA algorithm, aligning with recent TTA advancements [34]. We posit that the atomic feature-extraction components encoded by the branch-specific basis banks $\{\mathcal{B}^{(r)}\}_{r\in \mathcal{R}}$ are shared across tissue domains, whereas tissue-dependent differences are represented by their common mixing coefficient vector. Consequently, during the inference phase, we freeze all backbone parameters (including basis kernels and the router) and treat only the context embedding $z_{target}$ as the optimization variable. Given a labeled support set $\mathcal{S}=\{(x_{i},y_{i})\}_{i=1}^{N}$ from an unseen target tissue, where *N=50* in the standard setting, the target context embedding is optimized according to


(9) \begin{align*}& \begin{aligned} z_{\mathrm{target}}^{*} &= \arg\min_{z}\; \frac{1}{N} \sum_{(x,y)\in\mathcal{S}} \mathcal{L}_{\mathrm{MSE}} \left( \widehat{y}(x,z), y \right)\kern-2pt, \\ \widehat{y}(x,z) &= F \left( x; \left\{ \widetilde{W}^{(r)}(z) \right\}_{r\in\mathcal{R}} \right)\kern-2pt. \end{aligned}\end{align*}

Here, $\widehat{y}(x,z)$ denotes the prediction generated for sequence *x* using the branch-specific dynamic kernels synthesized from context embedding *z*. $\mathcal{S}$ denotes the labeled support set from the unseen target tissue; *N* is the support-set size; *x* and *y* denote an mRNA sequence and its corresponding ground-truth log-TE value; $\{\widetilde{W}^{(r)}(z)\}_{r\in \mathcal{R}}$ is the collection of the three branch-specific dynamic kernels generated from context embedding *z*; *F(· )* denotes the complete prediction network; and $\mathcal{L}_{\mathrm{MSE}}$ is the Mean Squared Error loss.

This process searches for a target-tissue coordinate on the pre-trained parameter manifold. Because only the low-dimensional context vector is optimized, it substantially reduces the number of trainable target-domain parameters relative to full fine-tuning and provides a parameter-efficient adaptation mechanism with a reduced risk of overfitting.

To further separate the effect of target-domain supervision from the choice of adaptation parameterization, we conducted matched adaptation-strategy controls using the same pre-trained ContiTE checkpoint and identical target-domain support/query partitions. The evaluated strategies included mean-source zero-shot inference, regression-head-only tuning, joint context-and-head tuning, full-parameter fine-tuning, and the proposed context-only TTA. For all adapted settings, *N=50* support samples and 50 optimization steps were used. Context-only TTA optimized only the 32-dimensional target context embedding with a learning rate of $1\times 10^{-2}$. For joint context-and-head tuning, the target context and regression head were optimized using learning rates of $1\times 10^{-2}$ and $1\times 10^{-3}$, respectively. The learning rates for head-only tuning and full fine-tuning were selected using validation tissues. Full implementation details are provided in the Supplementary Information.

To provide external baseline models with a directly comparable few-shot adaptation opportunity, we additionally evaluated RiboNN and mRNABERT under a matched output-head tuning protocol. Each baseline was first trained on the same source tissues used for ContiTE, without accessing any test tissue during source-domain training or model selection. For each unseen target tissue, the sequence encoder was frozen, and only the final regression head was optimized using the same *N=50* support samples, identical support/query partitions, and 50 adaptation steps used by ContiTE. The adaptation learning rate was selected from $\{1\times 10^{-4},1\times 10^{-3},1\times 10^{-2}\}$ using only the validation tissues. The experiments were repeated across the same five random seeds used in the matched adaptation-strategy analysis.

## Results

### Comparison with state-of-the-art methods

**Figure 4:**
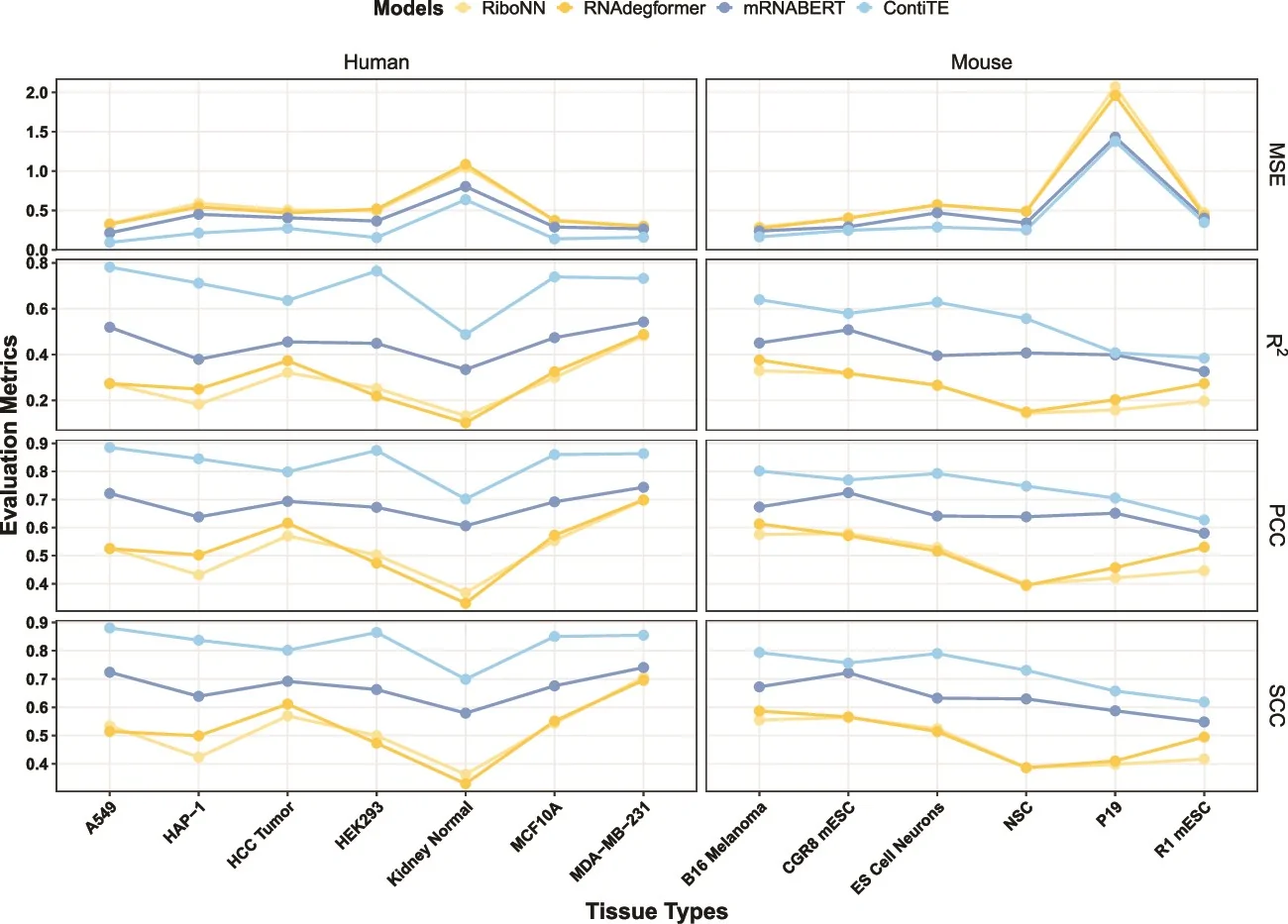
Performance comparison of ContiTE and three baseline models across seven Human and six Mouse target tissues. The four rows report MSE, $R^{2}$, PCC, and SCC, respectively. Lower values indicate better performance for MSE, whereas higher values indicate better performance for $R^{2}$, PCC, and SCC.

To comprehensively benchmark ContiTE’s capabilities in integrating multi-scale features and mitigating cross-tissue concept shift, we systematically compared it against three representative categories of state-of-the-art methods on an independent test set (Fig. 4). We first selected RiboNN, a deep CNN-based architecture, as the primary benchmark for capturing local cis-regulatory motifs. To extend the evaluation to global context modeling, we incorporated the Transformer-based RNAdegformer [17] to assess the efficacy of capturing long-range dependencies. Furthermore, we employed mRNABERT [18], a large-scale pre-trained language model, as a strong baseline to evaluate the transferability of universal self-supervised representations. This hierarchical comparison framework spans from local motif detectors to global sequence modelers and pre-trained universal encoders.

Notably, Fig. 4 evaluates practical predictive performance under a baseline-favorable target-domain supervision protocol: RiboNN, RNAdegformer, and mRNABERT were trained or fully fine-tuned using the labeled data available for each target tissue, whereas ContiTE adapted only a low-dimensional context embedding using *N=50* labeled target-domain samples. Because this benchmark differs in supervision budget and adaptation parameterization, it is not used alone to attribute the performance gain specifically to the weight-space mixing mechanism. We therefore conducted three complementary controls: matched adaptation-strategy comparisons within the same pre-trained ContiTE architecture, output-head tuning of RiboNN and mRNABERT using the same *N=50* target-domain support samples, and the w/o W-Space variant representing a conventional output-space MoE under a matched adaptation budget.

**Comparison with specialized deep learning models:** ContiTE demonstrates a dominant advantage over RNA-specific models (RNAdegformer, RiboNN). While baselines capture partial sequence features, they struggle with heterogeneous environmental regulation. Specifically, in human HAP-1 cells, ContiTE achieved an $R^{2}$ of 0.7114, surpassing RiboNN (*0.1826*) and RNAdegformer (*0.2487*) by 289% and 186%, respectively. ContiTE also realized a qualitative leap in Pearson correlation coefficient (PCC) (0.8451 versus 0.5022). These results show that ContiTE provides stronger predictive performance than the evaluated static sequence models under the present benchmark protocol.

**Comparison with large-scale pre-trained models:** We further benchmarked ContiTE against mRNABERT, a pre-trained SOTA model. Despite mRNABERT’s full fine-tuning, ContiTE achieved superior performance with minimal adaptation. For instance, in A549 cells, ContiTE improved $R^{2}$ by 50.6% (0.7817 versus 0.5190) and reduced MSE by 56.2%. This highlights the Data Efficiency and Positive Transfer of our “Atomization and Reconstruction” architecture. These results suggest that learning shared parameter-space components can provide stronger cross-tissue transferability than tissue-specific full fine-tuning under the evaluated setting. Together, the HAP-1 and A549 results provide representative examples of tissue-specific mRNA TE prediction in unseen target contexts. The reported metrics were calculated over the complete held-out mRNA query set of each tissue rather than over selectively chosen individual transcripts, thereby providing a more comprehensive assessment while avoiding potential cherry-picking of isolated examples.

**Superior ranking capability:** ContiTE exhibits strong ranking capability across the evaluated target tissues, with average PCC values of 0.8254 and 0.7355 on the human and mouse atlases, respectively, and performance exceeding 0.88 in the best-performing settings (e.g. HEK293 and A549). This ability to preserve the relative ordering of mRNA translation-efficiency values is important for applications such as mRNA candidate prioritization, where ranking may be more informative than absolute fitting.

### Ablation study

**Figure 5:**
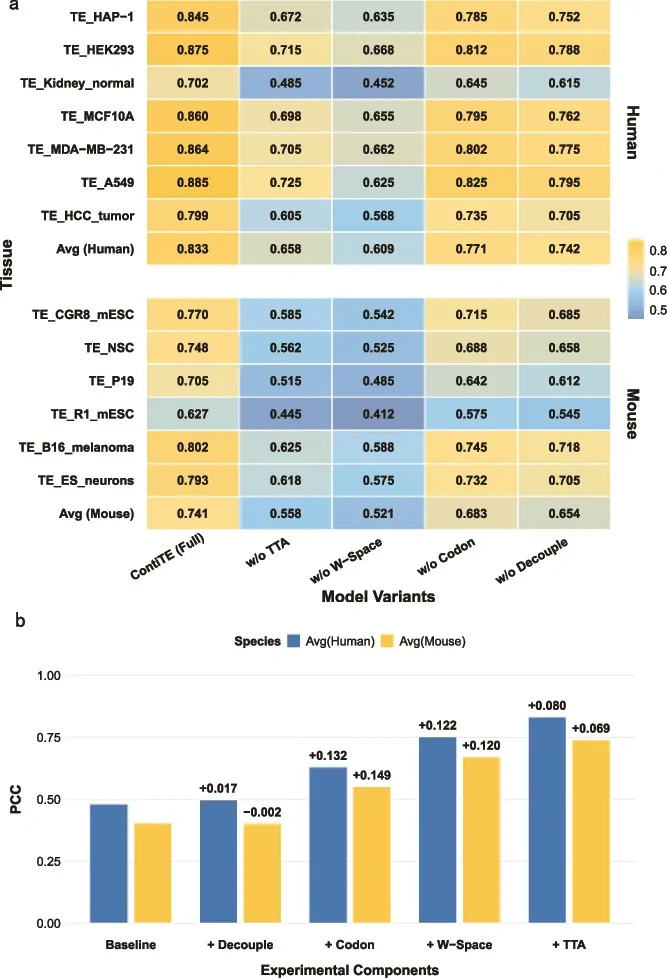
Ablation study of the ContiTE architecture on unseen human and mouse tissues. (a) Heatmap showing the PCC values of the full ContiTE model and its ablated variants across individual target tissues. The w/o TTA variant removes gradient-based target-domain context optimization according to the original ablation configuration; w/o W-Space replaces weight-space kernel synthesis with conventional output-space expert aggregation; w/o Codon replaces the codon-aware CDS convolution with a standard stride-1 convolution; and w/o Decouple removes the separate processing of the 5$^\prime $ UTR, CDS, and 3$^\prime $ UTR regions. (b) Progressive changes in average PCC across human and mouse tissues as the individual components are introduced into the baseline architecture. The annotated values indicate the incremental PCC changes contributed by each component.

To quantitatively evaluate the contribution of each core component within the ContiTE architecture and validate the effectiveness of our proposed “weight-space parameter reconstruction” and “biophysical inductive bias” in resolving concept shift, we conducted a series of rigorous ablation studies on unseen tissues (target domains) from both Human and Mouse datasets. The results are summarized in Fig. 5a. To further illustrate the marginal utility of each module, Fig. 5(b) depicts the progressive performance improvement as components are added sequentially to the baseline.

**Contribution of the adaptation strategy:** We compared the full model with the w/o TTA variant to quantify the contribution of the original target-domain adaptation module. In this ablation variant, gradient-based target-domain context optimization was removed according to the original ablation configuration. The w/o TTA variant achieved average PCC values of 0.658 and 0.558 on the human and mouse test tissues, respectively, compared with 0.833 and 0.741 for the full model. The performance decrease was particularly pronounced for Human Kidney and Mouse R1_mESC, for which PCC decreased from 0.702 to 0.485 and from 0.627 to 0.445, respectively. These results demonstrate that target-domain gradient calibration contributes substantially to the performance of the complete ContiTE framework. Importantly, the original w/o TTA ablation is distinct from the mean-source zero-shot inference control introduced in the additional matched adaptation-strategy experiments.

**Matched adaptation-strategy controls:** To determine whether the observed improvement was merely caused by access to labeled target-domain samples, we compared context-only TTA with mean-source zero-shot inference, regression-head-only tuning, joint context-and-head tuning, and full-parameter fine-tuning. All strategies used the same pretrained checkpoint and identical *N=50* support/query partitions across five random seeds. Context-only TTA achieved the highest average PCC on both the human and mouse atlases, reaching 0.8254 and 0.7355, respectively. Across the 13 target tissues, its average PCC exceeded mean-source zero-shot inference, head-only tuning, context-and-head tuning, and full fine-tuning by 0.0327, 0.0236, 0.0424, and 0.0283, respectively. All four comparisons remained significant after Holm correction ($P_{\mathrm{adj}}=.000977$). These within-architecture controls indicate that the benefit of ContiTE is not explained solely by exposure to target-domain supervision, but is associated with restricting adaptation to the low-dimensional context coordinate. Detailed results are provided in Supplementary Fig. S4 and Supplementary Table S4.

**External few-shot baseline controls:** We further provided RiboNN and mRNABERT with the same target-domain supervision available to ContiTE by tuning only their regression heads using identical *N=50* support/query partitions. This matched comparison directly controls for target-domain sample availability and complements the within-architecture adaptation controls and the output-space MoE ablation. Detailed performance and paired statistical comparisons are reported in Supplementary Table S11.

**Superiority of the weight-space paradigm:** To isolate the contribution of weight-space mixing from that of target-domain adaptation, we constructed the w/o W-Space variant as a conventional output-space MoE control. This variant retained the same sequence encoder, tissue split, support/query partition, support-set size (*N=50*), and adaptation-step budget as ContiTE, but replaced weight-space kernel synthesis with conventional output-space expert aggregation. During target-domain adaptation, its routing network was fine-tuned using the same support samples. The results indicate that this variant underperforms even the unadapted ContiTE in most test tissues, with an average PCC of only 0.609 on the Human dataset. In the sequential component analysis shown in Fig. 5b, introducing weight-space mixing produced incremental PCC gains of 0.122 and 0.120 for the human and mouse datasets, respectively. Taking Human A549 as an example, the standard MoE stagnated at a PCC of 0.625, far below ContiTE’s 0.885. This performance gap supports the advantage of weight-space mixing over conventional discrete output-space routing under the evaluated few-shot setting. Conversely, Weight-Space Mixing achieves continuous modeling of the parameter space via the linear span of basis kernels, thereby demonstrating superior robustness and expressivity.

**Effectiveness of the codon-periodicity-oriented inductive bias:** To evaluate the practical contribution of the CDS-branch configuration, we replaced the codon-periodicity-oriented convolution (Kernel*=3*, Stride*=3*) with a standard stride-1 convolution in the w/o Codon variant. Performance declined across all 13 target tissues, with an average PCC reduction of *∼ *0.06. Figure 5b further shows incremental PCC gains of 0.132 and 0.149 for the human and mouse atlases, respectively, after introducing the codon-oriented configuration. These results empirically support the utility of incorporating codon-scale triplet organization as an architectural inductive bias, but they do not establish a general signal-processing guarantee of phase invariance or noise suppression.

Removing the separate processing of the 5$^\prime $ UTR, CDS, and 3$^\prime $ UTR regions also reduced predictive performance. This result supports the practical benefit of separately modeling the heterogeneous sequence characteristics of coding and non-coding regions under the evaluated setting.

### Interpretability and visualization analysis

**Figure 6:**
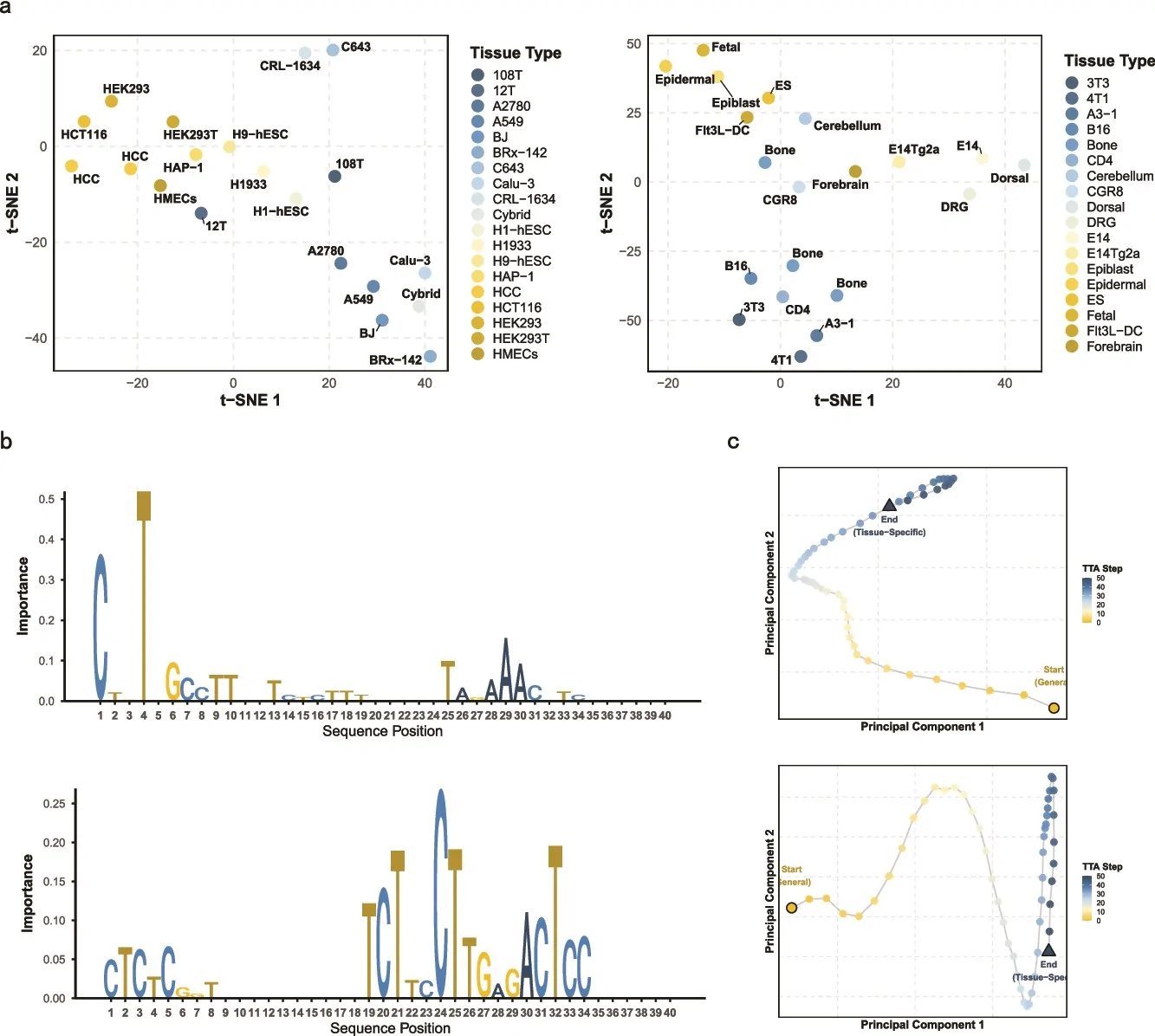
Visualization analyses of ContiTE. (a) Two-dimensional t-SNE projections of tissue-specific mixing coefficients for human and mouse tissue or cell contexts. Each point represents one context, and geometric proximity reflects similarity in the learned latent representation rather than direct molecular equivalence. (b) Integrated-Gradients attribution logos for representative 5$^\prime $ UTR sequences, where letter height denotes the magnitude of sequence-position attribution. (c) Principal-component projections of the target-context optimization trajectories over 50 TTA steps, from the mean source-tissue initialization to the adapted tissue-specific state. These analyses provide qualitative evidence of latent tissue organization, sequence-level attribution, and context calibration, but do not establish a one-to-one correspondence between individual basis kernels and specific trans-regulatory factors.

To empirically decipher the working mechanism of ContiTE, we present visualization analyses in Fig. 6 from three dimensions: latent manifold distribution, sequence motifs, and adaptation dynamics.

**The latent manifold of translational regulation:** We extracted the mixing coefficients *α * output by the Hyper-Router and projected them onto a 2D plane via t-SNE [35]. The results reveal that the tissue representations learned by ContiTE exhibit significant biological clustering properties (Fig. 6a). Observing the scatter plots, distinct tissues are not randomly distributed but spontaneously form well-demarcated clusters based on functional similarity. For instance, in the Human dataset (Fig. 6a, left), embryonic stem cell lines (H1-hESC and H9-hESC) and HCC samples are closely adjacent in the central region of the manifold space, forming a dense cluster representing a “high-proliferation/dedifferentiation” state. Conversely, in the Mouse dataset (Fig. 6a, right), pluripotent stem cell lines like ES and CGR8 cluster on the left, while differentiated tissues such as Bone and DRG are distributed on the right and bottom, completely separated from the stem cell lines. This topological structure supports the view that the learned tissue representations organize biologically related contexts in a continuous latent space rather than merely memorizing discrete tissue labels.

**Dynamic perception of sequence motifs:** Sequence Logos of the 5$^\prime $ UTR region, generated via the Integrated Gradients algorithm [36], reveal the model’s acute capture of key cis-regulatory elements (Fig. 6b). Visually, the sequence importance scores are not uniformly distributed but exhibit strong sparsity and a high signal-to-noise ratio. In the region near the CDS start site at the 3$^\prime $ end of the sequence, several giant base characters (e.g. the towering “T” and “C” in Fig. 6b, bottom) stand out abruptly, with heights far exceeding the background noise. This striking visual contrast indicates that ContiTE has learned a focus strategy akin to an attention mechanism within specific tissue contexts. It effectively suppresses non-critical background sequences and precisely locks onto key features governing translation initiation efficiency, such as the core positions of the Kozak sequence, suggesting that the model can adjust its sequence-level attribution patterns across tissue contexts.

**Trajectory of data-efficient adaptation:** The visualization of the optimization path of the context vector *z* during the TTA phase demonstrates the model’s efficient transfer capability (Fig. 6c). The plot depicts a trajectory connecting the general initialization state (yellow circle, “Start”) and the tissue-specific state (dark blue triangle, “End”), with points colored according to the TTA steps. The trajectory presents a smooth, loop-free arc shape with a relatively clear path. This optimization dynamics suggests that the TTA algorithm provides explicit and efficient gradient guidance in the principal component space, allowing the model to bridge the gap from “Generalist” to “Specialist” with minimal update steps (few-shot steps). This optimization trajectory provides qualitative support for the efficient context calibration observed during few-shot target-domain adaptation.

### Data efficiency and sensitivity analysis

**Figure 7:**
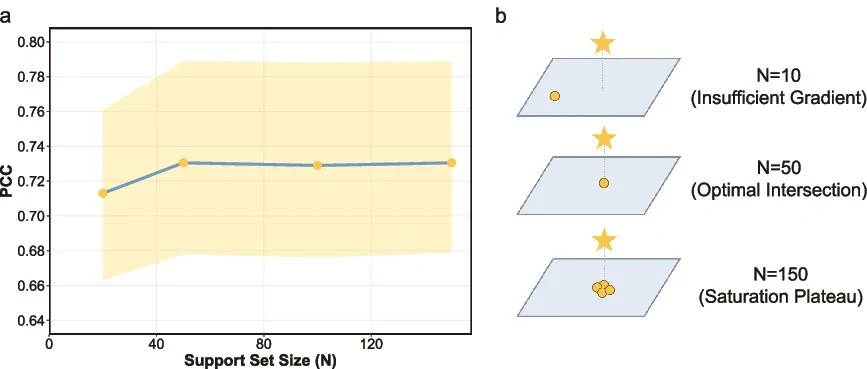
Sensitivity of ContiTE to target-domain support-set size. (a) Average PCC across the evaluated target tissues at support-set sizes of *N=10*, *50*, *100*, and *150*. Performance improves markedly from *N=10* to *N=50*, whereas only modest additional changes are observed at *N=100* and *N=150*. (b) Schematic summary of the three observed data regimes: limited supervision at *N=10*, the selected operating point at *N=50*, and diminishing marginal gains at larger support-set sizes.

The performance of TTA depends on the availability of labeled samples from the target domain. To characterize adaptation under different levels of target-domain supervision, we evaluated ContiTE using support-set sizes of $N=\{10,50,100,150\}$ while keeping the remaining adaptation settings unchanged. The results are shown in Fig. 7. At *N=10*, ContiTE retained non-trivial predictive performance, but the limited supervision resulted in a lower average PCC and greater variation across target tissues. Increasing the support set to *N=50* produced a clear improvement in average performance. Further increasing the support size to *N=100* and *N=150* resulted in only minor changes, indicating diminishing marginal gains within the evaluated range. We therefore selected *N=50* as the standard operating point in the main experiments because it provided a practical balance between predictive performance and target-domain annotation requirements. This selection is specific to the evaluated atlas and should not be interpreted as a universally optimal support-set size for all rare tissues or clinical samples. Taken together, these results identify *N=50* as a suitable operating point for the present benchmark, providing substantially better performance than *N=10* while showing only limited additional gains at *N=100* and *N=150*. The analysis demonstrates the relative sample efficiency of context-embedding adaptation within the evaluated datasets, but it does not imply that 50 samples are universally sufficient or that overfitting is impossible in more heterogeneous clinical settings.

We further examined more stringent adaptation regimes using mean-source zero-shot inference and support-set sizes of *N=1*, *5*, *20*, and *50* across five random seeds. Average PCC increased from 0.7962 under mean-source zero-shot inference to 0.8254 at *N=50* for the human atlas, and from 0.6987 to 0.7355 for the mouse atlas. Single-sample adaptation showed greater variability and was slightly less stable than mean-source zero-shot inference, whereas improvements became more consistent at *N=20* and *N=50*. Because the query set changes with the support size, this analysis is interpreted as an overall data-efficiency trend rather than as a strictly paired comparison between adjacent support sizes. Detailed results are provided in Supplementary Fig. S9 and Supplementary Table S9.

We additionally evaluated the sensitivity to the number of atomic basis kernels on the human atlas. Under a single-run computational sensitivity protocol, *K=4* achieved the highest validation and test PCC values (0.7627 and 0.8254), compared with 0.6980 and 0.7950 for *K=2*, and 0.7471 and 0.8138 for *K=8*. Thus, *K=4* provided the best performance under the evaluated protocol, while increasing the basis number to eight did not yield further improvement. Because the analysis was restricted to the human atlas and a single training run, it should be interpreted as a computational sensitivity analysis rather than a multi-seed or cross-species hyperparameter benchmark. Detailed results are provided in Supplementary Fig. S8 and Supplementary Table S8.

## Discussion and conclusion

In this study, we introduced ContiTE, a parameter-efficient few-shot adaptation framework explicitly designed to address the pervasive challenge of concept shift in cross-tissue mRNA TE prediction. By synergizing the MoE mechanism with continuous parameter generation, ContiTE transitions the paradigm from traditional discrete output-space routing to continuous weight-space mixing. Coupled with biologically informed structural designs and a gradient-based TTA strategy, our framework achieved breakthrough predictive performance on unseen tissues across both human and mouse atlases, demonstrating an effective parameter-reconstruction strategy for few-shot cross-tissue mRNA translation-efficiency prediction.

The performance of ContiTE can be attributed to three complementary architectural choices. First, separately parameterized basis banks for the 5$^\prime $ UTR, CDS, and 3$^\prime $ UTR branches allow the same tissue-conditioned coordinate to reconstruct region-specific feature extractors while preserving the heterogeneous sequence characteristics of the three functional regions. Second, the Kernel*=3*, Stride*=3* CDS configuration introduces a codon-scale architectural inductive bias. Its utility is supported by the w/o Codon ablation, although this configuration should not be interpreted as a mathematical guarantee of phase invariance or noise suppression. Third, context-only TTA restricts target-domain adaptation to a low-dimensional coordinate, providing a parameter-efficient alternative to full-network fine-tuning.

From a biological perspective, the learned tissue representations suggest that translational regulatory contexts are not organized as completely isolated categories. The proximity of several related pluripotent, differentiated, and cancer-associated contexts is consistent with partially shared translational programs, although latent-space proximity alone cannot identify the responsible molecular regulators. The 5$^\prime $ UTR Integrated-Gradients analysis further indicates that predictive importance is concentrated within restricted sequence regions rather than being uniformly distributed across the input, supporting the relevance of localized cis-regulatory information for tissue-dependent TE prediction. Nevertheless, these attribution patterns are model-derived hypotheses and should not be interpreted as experimentally validated novel motifs or as evidence that individual basis kernels correspond to specific trans-acting factors.

An important future direction is to augment the freely learned tissue context embedding with experimentally measured molecular-context information. Tissue-specific tRNA abundance or tRNA adaptation index profiles could provide information on codon-dependent decoding capacity; RBP expression and eCLIP-derived occupancy profiles could represent transcript-specific post-transcriptional regulation; and epigenetic or epitranscriptomic measurements could provide complementary information on cellular regulatory state. These heterogeneous signals could be encoded by a dedicated context encoder and fused with the learned tissue embedding before the Context Hyper-Router. Such multimodal conditioning may improve biological identifiability and potentially reduce the amount of labeled target-domain TE data required for adaptation, but it will require matched multi-omics measurements across tissues.

The present study also inherits limitations from the underlying RiboNN resource and its processed Ribo-seq and RNA-seq measurements. Ribo-seq-derived TE estimates may be affected by protocol-specific footprint recovery, nuclease digestion, library preparation, read mapping, transcript-abundance estimation, and batch-dependent variation. Moreover, although the evaluated atlas includes pluripotent cells, differentiated states, immortalized cell lines, and cancer-associated samples, it does not comprehensively represent primary cells, clinical specimens, or in vivo tissue environments. Consequently, generalization to these settings should be evaluated using independently generated datasets and, ideally, prospective experimental validation.

Despite these advances, several limitations define the current scope of ContiTE. First, the present weight-synthesis mechanism constructs tissue-specific parameters through convex combinations of the learned basis kernels and is therefore expected to be most reliable when a target tissue remains within, or close to, the regulatory variation represented by the source atlas. A post-hoc analysis across the 13 target tissues did not identify a significant monotonic association between source-manifold distance and absolute TTA performance (Spearman *ρ =-0.2473*, permutation *P=.4192*). However, more distant target tissues exhibited larger adaptation gains over mean-source zero-shot inference in the combined analysis (*ρ =0.6758*, permutation *P=.0127*, bootstrap 95% CI: 0.2605–0.8592). Because the associations were not significant when the human and mouse tissues were analyzed separately, this result should be interpreted as exploratory evidence from a pooled analysis of two species-specific cross-tissue benchmarks rather than as evidence of a species-independent relationship. The present experiments also do not establish the behavior of ContiTE under extreme pathological or stress-induced out-of-distribution states. Second, the current adaptation mechanism still requires labeled translation-efficiency measurements from the target tissue. Initialization analyses showed that source-mean, zero-vector, and support-aware best-source initialization produced comparable final performance, whereas random Gaussian and randomly selected source initialization reduced average PCC by *∼ *0.015 and 0.014, respectively. The model was minimally affected by simulated support-label noise up to a relative level of 0.3. Tissue contamination produced only modest degradation, although 20% and 30% contamination caused small but statistically detectable PCC reductions in the human atlas. Mean-source zero-shot and extremely low-sample analyses further showed that single-sample adaptation can be unstable, whereas performance improvements become more consistent with 20–50 labeled samples. These computational perturbation experiments do not reproduce all characteristics of low-depth sequencing, heterogeneous clinical biopsies, or experimental batch effects, and validation in such settings remains necessary. Finally, the atomic basis kernels should be interpreted as latent parameter-space components rather than as direct measurements of individual trans-regulatory factors. A representative sensitivity analysis on the human atlas favored *K=4* over *K=2* and *K=8* under the evaluated training protocol. Cosine-similarity analysis further revealed branch-specific differences in basis diversity and redundancy: the CDS branch exhibited lower inter-basis similarity than the UTR branches, and strict orthogonality was neither observed nor assumed. Because the *K* sensitivity analysis was limited to a single training run on the human atlas, broader multi-seed evaluation in both species and dedicated cross-species transfer experiments remain warranted. Moreover, the empirical validation is currently restricted to cross-tissue mRNA translation-efficiency prediction, and extension to other scientific learning tasks will require task-specific validation.

**Key Points**

- ContiTE proposes a continuous manifold mixture-of-experts framework to address biological concept shift in cross-tissue mRNA translation efficiency prediction.
- A novel weight-space mixing paradigm dynamically synthesizes domain-specific weights, coupled with a highly efficient TTA strategy for rapid few-shot transfer to unseen tissues.
- ContiTE achieves state-of-the-art performance on comprehensive human and mouse atlases (improving average PCC by 12.09%), while offering deep interpretability of the latent regulatory manifold.

## Supplementary Material

SI-ContiTE_final_bbag518

## Data Availability

The code and dataset can be accessed at: https://github.com/wyx2012/conTi_Te_.
