# Supplementary material for: ContiTE: continuous manifold MoE for few-shot cross-tissue mRNA translation efficiency prediction
Source: Brief Bioinform. 2026 Sep 21;27(5):bbag518. doi: 10.1093/bib/bbag518 (PMC13591282; doi:10.1093/bib/bbag518)
Supplement: Supplementary file 1 [file si-contite_final_bbag518.pdf]

Briefings in Bioinformatics, 2026, pp. 1–17
DOI added during production
Published: Date added during production
Original Paper

Supplementary Information ContiTE: Continuous Manifold MoE for Few-Shot Cross-Tissue mRNA Translation Efficiency Prediction

Yuxiao Wei,[1] Qi Zhang,[2] Sainan Huo[1] and Xuezhong Zhou[1,*]

[1]School of Computer Science & Technology, Beijing Jiaotong University, Beijing, 100044, China
[2]School of Computer Science and Engineering, Nanjing University of Science and Technology, 200 Xiaolingwei, Nanjing, 210094, China

*Corresponding author. xzzhou@bjtu.edu.cn

Abstract

Predicting mRNA Translation Efficiency (TE) across diverse tissues is a critical yet challenging task due to the phenomenon of concept shift, where identical genomic sequences exhibit distinct functional profiles across varying cellular environments. Existing static models are often limited by fixed parameterization, struggling to capture these continuous regulatory variations without requiring computationally expensive fine-tuning. To address these limitations, we propose ContiTE, a continuous manifold Mixture-of-Experts (MoE) framework explicitly designed for efficient few-shot cross-tissue adaptation. Unlike traditional MoE architectures that rely on discrete output-space mixing, ContiTE operates on a continuous parameter manifold by dynamically synthesizing domain-specific weights through a context-aware hyper-router that linearly combines shared atomic basis kernels. This paradigm enables smooth interpolation of translational rules and provides a flexible mechanism to model complex, context-dependent biological regulation. Furthermore, we introduce a gradient-based Test-Time Adaptation (TTA) strategy that allows the model to rapidly align to new, unseen tissues by solely optimizing low-dimensional context embeddings while keeping the backbone parameters frozen. Experimental results on a comprehensive human and mouse atlas demonstrate that ContiTE significantly outperforms state-of-the-art methods in few-shot scenarios, improving average Pearson Correlation Coefficient (PCC) by 12.09% and R^2 by 18.22%. By mitigating negative transfer and requiring only limited target-domain data for calibration, ContiTE provides a computational framework for tissue-specific mRNA translation-efficiency prediction and demonstrates a parameter-reconstruction strategy that may be extensible to other cross-domain sequence-learning tasks, subject to task-specific validation.

Keywords mRNA translation efficiency, continuous manifold, mixture of experts, few-shot adaptation, test-time adaptation

Theoretical Analysis

In this section, we provide a rigorous mathematical formulation of the ContiTE framework, contrasting it with the standard Mixture-of-Experts (MoE) paradigm. We further analyze the theoretical justification for the Weight-Space Mixing mechanism and derive the gradient dynamics that enable efficient Test-Time Adaptation (TTA).

Notations and Problem Definition

Let $\mathcal{D} = \{(\mathcal{X}_d, \mathcal{Y}_d)\}_{d=1}^{M}$ denote a set of M source domains (tissues), where each domain follows a distinct data distribution $P_d(\mathbf{X}, y)$.

- **Sequence Branches:** Let $\mathcal{R} = \{5'\mathrm{UTR}, \mathrm{CDS}, 3'\mathrm{UTR}\}$ denote the three functional sequence branches.
- **Input Space:** For each branch $r \in \mathcal{R}$, let $\mathbf{X}^{(r)} \in \mathbb{R}^{C_{\mathrm{in}} \times L_r}$ denote the embedded multi-channel sequence representation, where L_r is the branch-specific sequence length and C_{in} is the number of input channels.
- **Output Space:** Let $y \in \mathbb{R}$ be the scalar Translation Efficiency (TE) value.
- **Branch-specific Basis Banks:** For each branch $r \in \mathcal{R}$, let $\mathcal{B}^{(r)} = \{\mathbf{B}_1^{(r)}, \dots, \mathbf{B}_K^{(r)}\}$, where $\mathbf{B}_k^{(r)} \in \mathbb{R}^{C_{\mathrm{out}} \times C_{\mathrm{in}} \times S_r}$. Here, S_r is the branch-specific kernel size. The complete basis collection is denoted by $\mathcal{B} = \{\mathcal{B}^{(r)}\}_{r \in \mathcal{R}}$. The three branches maintain separately parameterized basis banks because they use different convolutional configurations, whereas the basis kernels within each branch are shared across tissue domains.
- **Context Space:** Let $\mathbf{z}_d \in \mathbb{R}^{D_z}$ be the latent context embedding corresponding to tissue domain d.

Contrast: Discrete Output-Space vs. Continuous Weight-Space Manifold Mixing

Definition 1.1 (Standard Output-Space MoE). In a traditional MoE layer, the input $\mathbf{X}$ is processed by K independent expert networks $\{E_k\}_{k=1}^{K}$. A gating network $G(\mathbf{X})$ determines the routing weights.

© The Author(s) 2026. Copyright and licence statements to be updated by the publisher during production.

The output $\mathbf{Y}_{out}$ is the weighted sum of expert *outputs*:

$$\mathbf{Y}_{out} = \sum_{k=1}^{K} [G(\mathbf{X})]_k \cdot E_k(\mathbf{X}; \theta_k) \tag{1}$$

where θ_k represents fixed parameters for the k-th expert.

Limitation: The experts are discrete and fixed functions. Adaptation requires changing the routing $G(\mathbf{X})$, which is often sparse and discontinuous, making it difficult to model subtle shifts in biological manifolds.

Definition 1.2 (**ContiTE: Weight-Space Mixing**)**.** ContiTE treats the convolutional weights as functions of the tissue context embedding $\mathbf{z}_d$. Let $\Phi : \mathbb{R}^{D_z} \to \Delta^{K-1}$ denote the Context Hyper-Router. It produces the tissue-conditioned mixing coefficients $\boldsymbol{\alpha}_d = \Phi(\mathbf{z}_d)$, where $\boldsymbol{\alpha}_d \in \mathbb{R}^K$, $\alpha_{d,k} \geq 0$, and $\sum_{k=1}^{K} \alpha_{d,k} = 1$.

For each sequence branch $r \in \mathcal{R}$, the corresponding domain-specific dynamic convolutional kernel is synthesized from its branch-specific basis bank using the same tissue-conditioned coefficient vector:

$$\mathbf{W}_d^{(r)}(\mathbf{z}_d) = \sum_{k=1}^{K} \alpha_{d,k} \mathbf{B}_k^{(r)}, \qquad r \in \mathcal{R}. \tag{2}$$

Using the cross-correlation convention implemented by standard deep-learning libraries, the multi-channel one-dimensional convolution for branch r and its linearity with respect to the synthesized kernel are expressed as follows:

$$\begin{aligned} U_{d,o,t}^{(r)} &= b_o^{(r)} + \sum_{c=1}^{C_{\text{in}}} \sum_{j=0}^{S_r - 1} W_{d,o,c,j}^{(r)} X_{c,ts_r+j}^{(r)}, \\ H_{d,o,t}^{(r)} &= \sigma\left(U_{d,o,t}^{(r)}\right). \end{aligned} \tag{3a}$$

$$\mathbf{X}^{(r)} \star \left(\sum_{k=1}^{K} \alpha_{d,k} \mathbf{B}_k^{(r)} \right) = \sum_{k=1}^{K} \alpha_{d,k} \left(\mathbf{X}^{(r)} \star \mathbf{B}_k^{(r)} \right). \tag{3b}$$

Here, o and c index the output and input channels, respectively; j indexes the kernel position; t indexes the convolutional output position; S_r and s_r are the branch-specific kernel size and stride; $b_o^{(r)}$ is the bias associated with output channel o; and $\sigma(\cdot)$ denotes the nonlinear activation function. The symbol $\star$ denotes the cross-correlation operation used by the implementation.

Advantage: Equation (3b) follows from the linearity of cross-correlation with respect to the kernel weights. Therefore, changing $\mathbf{z}_d$ continuously changes the synthesized branch-specific kernels within the convex hull of the learned basis kernels.

Manifold Assumption and Meta-Learning Perspective

ContiTE can be viewed as a meta-learning framework where the context $\mathbf{z}_d$ parameterizes the task distribution.

Assumption 1.3 (**Low-Rank Parameter Manifold**)**.** We assume that the tissue-dependent dynamic convolutional parameters do not occupy the entire high-dimensional parameter space. Instead, the branch-specific optimal kernels can be approximated using a shared low-dimensional coefficient vector over the learned basis banks:

$$\begin{aligned} \left\{ \mathbf{W}_d^{(r)*} \right\}_{r \in \mathcal{R}} \approx \Bigg\{ \left\{ \sum_{k=1}^{K} \alpha_{d,k} \mathbf{B}_k^{(r)} \right\}_{r \in \mathcal{R}} : \\ \boldsymbol{\alpha}_d \in \Delta^{K-1} \Bigg\}. \end{aligned} \tag{4}$$

Under this assumption, meta-training jointly learns the branch-specific basis banks, the Context Hyper-Router, and the remaining encoder parameters by minimizing the expected source-domain risk:

$$\begin{aligned} &\min_{\mathcal{B}, \Phi, \Theta_{\text{enc}}} \quad \mathbb{E}_{d \sim \mathcal{D}} \left[\frac{1}{|D_d|} \sum_{(\mathbf{x}, y) \in D_d} \mathcal{L}\left(\widehat{y}_d(\mathbf{x}), y\right) \right], \\ &\widehat{y}_d(\mathbf{x}) = F\left(\mathbf{x}; \left\{ \mathbf{W}_d^{(r)}(\mathbf{z}_d) \right\}_{r \in \mathcal{R}} \right). \end{aligned} \tag{5}$$

Here, $\widehat{y}_d(\mathbf{x})$ denotes the model prediction for input $\mathbf{x}$ in tissue domain d; D_d denotes the training samples of domain d; and Θ_{enc} contains the remaining trainable sequence encoder parameters.

Derivation of Test-Time Adaptation (TTA) Dynamics

A key contribution of ContiTE is gradient-based adaptation of the low-dimensional context embedding. For a single sequence–label pair, the squared-error loss is defined as $\mathcal{L}_{\text{MSE}} = \frac{1}{2}(y - \widehat{y})^2$. During target-domain adaptation, all branch-specific basis banks, the Context Hyper-Router, the sequence encoder, the branch biases, and the regression head are frozen. Only $\mathbf{z}_d$ is optimized according to $\mathbf{z}_d \leftarrow \mathbf{z}_d - \eta \nabla_{\mathbf{z}_d} \mathcal{L}$, where η denotes the adaptation learning rate and $\nabla_{\mathbf{z}_d} \mathcal{L}$ is the gradient of the support-set loss with respect to the target context embedding.

For a rigorous treatment of the convolutional gradient, we use an unfolded sliding-window representation. For branch r, define

$$\overline{\mathbf{X}}^{(r)} = \text{Unfold}_{S_r, s_r}\left(\mathbf{X}^{(r)}\right) \in \mathbb{R}^{(C_{\text{in}} S_r) \times T_r}, \tag{6a}$$

$$\mathbf{U}_d^{(r)} = \overline{\mathbf{W}}_d^{(r)} \overline{\mathbf{X}}^{(r)} + \mathbf{b}^{(r)} \mathbf{1}_{T_r}^{\top}, \tag{6b}$$

$$\frac{\partial \mathcal{L}}{\partial \overline{\mathbf{W}}_d^{(r)}} = \boldsymbol{\Delta}^{(r)} \left(\overline{\mathbf{X}}^{(r)}\right)^{\top}, \tag{6c}$$

$$\frac{\partial \mathcal{L}}{\partial \alpha_{d,k}} = \sum_{r \in \mathcal{R}} \left\langle \frac{\partial \mathcal{L}}{\partial \overline{\mathbf{W}}_d^{(r)}}, \overline{\mathbf{B}}_k^{(r)} \right\rangle_F, \tag{6d}$$

$$\frac{\partial \mathcal{L}}{\partial \mathbf{b}^{(r)}} = \boldsymbol{\Delta}^{(r)} \mathbf{1}_{T_r}. \tag{6e}$$

Here, $\overline{\mathbf{X}}^{(r)} \in \mathbb{R}^{(C_{\text{in}} S_r) \times T_r}$ is the unfolded input matrix whose columns contain the multi-channel sliding windows of branch r; S_r and s_r are the branch-specific kernel size and stride; and T_r is the number of convolutional output positions. The matrix $\overline{\mathbf{W}}_d^{(r)} \in \mathbb{R}^{C_{\text{out}} \times (C_{\text{in}} S_r)}$ is the reshaped dynamic kernel, while $\mathbf{U}_d^{(r)} \in \mathbb{R}^{C_{\text{out}} \times T_r}$ is the corresponding pre-activation output. Furthermore, $\mathbf{b}^{(r)} \in \mathbb{R}^{C_{\text{out}}}$ is the branch-specific bias vector, and $\mathbf{1}_{T_r} \in \mathbb{R}^{T_r}$ is the all-ones vector.

The upstream gradient is $\boldsymbol{\Delta}^{(r)} = \partial \mathcal{L} / \partial \mathbf{U}_d^{(r)} \in \mathbb{R}^{C_{\text{out}} \times T_r}$. It includes the gradient propagated through the nonlinear activation, global max pooling, branch concatenation, and regression head. The matrix $\overline{\mathbf{B}}_k^{(r)}$ is the reshaped form of the k-th atomic basis kernel of branch r; $\alpha_{d,k}$ is its tissue-conditioned mixing coefficient; and $\langle \cdot, \cdot \rangle_F$ denotes the Frobenius inner product.

Because $\boldsymbol{\alpha}_d = \Phi(\mathbf{z}_d)$, the context-embedding gradient is

$$\nabla_{\mathbf{z}_d}\mathcal{L} = J_\Phi(\mathbf{z}_d)^\top \nabla_{\boldsymbol{\alpha}_d}\mathcal{L}. \tag{7}$$

Here, $J_\Phi(\mathbf{z}_d) = \partial\boldsymbol{\alpha}_d/\partial\mathbf{z}_d$ is the Jacobian of the Context Hyper-Router; $\nabla_{\boldsymbol{\alpha}_d}\mathcal{L} \in \mathbb{R}^K$ is the gradient of the loss with respect to the complete mixing coefficient vector; and $\nabla_{\mathbf{z}_d}\mathcal{L} \in \mathbb{R}^{D_z}$ is the resulting gradient with respect to the tissue context embedding.

Although Equation (6e) gives the gradient with respect to the branch bias, the biases are frozen during context-only TTA and are not generated from $\mathbf{z}_d$. Consequently, this gradient does not contribute to the update of the target context embedding.

Theoretical Interpretation: The context gradient depends on three components: the upstream prediction error propagated through the complete downstream network, the multi-channel input windows involved in each convolution, and the alignment between the resulting convolutional-weight gradient and the branch-specific atomic basis kernels. The router Jacobian maps the coefficient-level gradients into the low-dimensional context space. This formulation preserves the parameter-efficiency interpretation of context-only TTA without treating a convolutional layer as a fully connected operation.

Conditional Capacity Analysis of the TTA Hypothesis Space

We analyze the effective capacity of ContiTE during target-domain adaptation using Rademacher complexity. This analysis is conditional on a model that has already completed meta-training. Although the branch-specific basis banks and Context Hyper-Router are jointly optimized during meta-training, they are frozen during TTA, and only the target-tissue context embedding is optimized. Therefore, the following analysis characterizes the restricted TTA hypothesis space and should not be interpreted as a convergence theorem for joint meta-training or as a tight prediction-error guarantee.

Definition 2.1 (TTA Hypothesis Space). Under the Test-Time Adaptation protocol, the encoder parameters, branch-specific basis banks, Context Hyper-Router, branch biases, and regression head are frozen. Only the tissue-specific context embedding $\mathbf{z}$ is optimized. The resulting hypothesis space is

$$\begin{aligned}\mathcal{H}_{\mathrm{TTA}} = \Bigg\{ f(\mathbf{x};\mathbf{z}) = F\left(\mathbf{x}; \left\{\mathbf{W}^{(r)}(\mathbf{z})\right\}_{r\in\mathcal{R}}\right) : \\ \mathbf{z}\in\mathbb{R}^{D_z}, \qquad \|\mathbf{z}\|_2 \le R_z \Bigg\}.\end{aligned} \tag{8}$$

Here, $\mathbf{W}^{(r)}(\mathbf{z}) = \sum_{k=1}^{K}[\Phi(\mathbf{z})]_k \mathbf{B}_k^{(r)}$ is the dynamic kernel synthesized for branch r; D_z is the dimensionality of the context embedding; R_z is the radius of the norm-bounded context set; and $F(\cdot)$ denotes the complete frozen predictor.

Theorem 2.2 (Conditional Generalization Bound for the TTA Stage). *Assume that the context embedding is restricted to the norm-bounded set defined in Equation* (8)*, that the composite predictor* $F(\mathbf{x};\{\mathbf{W}^{(r)}(\mathbf{z})\}_{r\in\mathcal{R}})$ *is Lipschitz continuous with respect to* $\mathbf{z}$ *over this set, and that the loss function* ℓ *is* L_ℓ*-Lipschitz and bounded by* M*. For an unseen tissue domain, with probability at least* $1-\delta$ *over a support set* $\mathcal{S}$ *of size* N*, the expected risk satisfies*

$$R(\mathbf{z}) \le \widehat{R}_{\mathcal{S}}(\mathbf{z}) + 2L_\ell \mathfrak{R}_N\left(\mathcal{H}_{\mathrm{TTA}}\right) + M\sqrt{\frac{\log(1/\delta)}{2N}}. \tag{9}$$

Here, $R(\mathbf{z})$ and $\widehat{R}_{\mathcal{S}}(\mathbf{z})$ denote the expected and empirical risks, respectively; $\mathfrak{R}_N(\mathcal{H}_{\mathrm{TTA}})$ is the Rademacher complexity of the restricted predictor class; L_ℓ is the Lipschitz constant of the loss; M is an upper bound on the loss; N is the support-set size; and $\delta \in (0,1)$ is the confidence parameter.

Theoretical Justification for Sample Efficiency

The efficiency of ContiTE stems from the reduction in the dimension of the target-domain optimization space. Standard full fine-tuning operates over all $P = 45{,}429$ trainable model parameters, whereas context-only TTA optimizes a D_z-dimensional context embedding. Under standard boundedness and Lipschitz assumptions, the capacity of this restricted parametric class exhibits the qualitative scaling

$$\mathfrak{R}_N\left(\mathcal{H}_{\mathrm{TTA}}\right) = O\left(C_{\mathrm{TTA}}\sqrt{\frac{D_z}{N}}\right). \tag{10}$$

Here, C_{TTA} collects constants associated with the norm bound of the context space and the Lipschitz sensitivity of the frozen predictor; D_z is the context-embedding dimension; and N is the support-set size. This expression describes qualitative capacity scaling under the stated assumptions rather than an exact equality for the trained network.

Because $D_z = 32 \ll P$, restricting adaptation to the context embedding substantially reduces the number of target-domain degrees of freedom relative to full-parameter fine-tuning. This reduced hypothesis space provides a qualitative explanation for the sample efficiency of context-only TTA, but it does not guarantee rapid optimization, convexity, or a small empirical error for every unseen tissue.

Basis Diversity and Manifold Coverage

The expressive capacity of ContiTE depends on whether the learned basis kernels provide sufficiently diverse and non-redundant directions in parameter space. In the current implementation, the basis kernels are jointly learned through the translation-efficiency prediction objective, without an explicit orthogonality constraint. Consequently, strict orthogonality is neither assumed nor guaranteed. The basis set should instead be interpreted as a compact collection of shared parameter components whose context-dependent combinations support tissue-specific weight synthesis. Explicit diversity-promoting or orthogonality regularization may be investigated in future work.

Dataset Sources and Accessibility

All experimental data used in this study were obtained from the processed human and mouse translation-efficiency atlases released with RiboNN. The human and mouse data are two species-specific subsets of the same RiboNN resource rather than independent external benchmark datasets. The human subset contains 11,798 genes across 96 cell or tissue contexts, whereas the mouse subset contains 11,575 genes across 46 contexts. The corresponding source files and direct download links are provided in Supplementary Table S1.

Table S1 Source information and accessibility of the datasets used in the ContiTE experiments.

Dataset Subset	Scale	Source File and Access Link
RiboNN Human atlas	11,798 genes across 96 cell/tissue contexts	MOESM3 (Download Human dataset)
RiboNN Mouse atlas	11,575 genes across 46 cell/tissue contexts	MOESM4 (Download Mouse dataset)

Both subsets were obtained from the processed supplementary data released with RiboNN. The exact tissue-level training, validation, and test partitions used in ContiTE are provided with the project code.

Table S2 Hyperparameter specifications of ContiTE.

Category	Hyperparameter	Value / Description
Data Processing	Vocabulary	$\{A, C, G, T, N\}$ mapped to $\{0, 1, 2, 3, 4\}$
	Max Length (5' UTR)	500 nt (Padding/Truncation)
	Max Length (CDS)	2000 nt (Padding/Truncation)
	Max Length (3' UTR)	1500 nt (Padding/Truncation)
Model Architecture	Sequence Embedding Dim	16
	Tissue Context Embedding Dim (D_z)	32
	Number of Basis Kernels (K)	4
	Dynamic Conv (5' UTR)	Kernel=7, Stride=1, Channels=16→32
	Dynamic Conv (CDS)	Kernel=3, Stride=3, Channels=16→32 (Periodicity Bias)
	Dynamic Conv (3' UTR)	Kernel=7, Stride=1, Channels=16→32
	Router MLP Hidden Dim	64
	Regressor Hidden Dim	64
Meta-Training	Optimizer	Adam
	Learning Rate	1×10^{-3}
	Batch Size	64
	Loss Function	Mean Squared Error (MSE)
	Max Epochs	200
	Early Stopping Patience	15 epochs (min. 50 epochs)
	LR Scheduler	ReduceLROnPlateau (Factor=0.5, Patience=5)
Test-Time Adaptation	Adaptation Optimizer	Adam
	Adaptation Learning Rate	1×10^{-2}
	Adaptation Steps	50
	Support Set Size	50 labeled samples (standard setting)
	Support Batch Size	16

All hyperparameters were selected based on preliminary experiments on the validation set.

The hyperparameter settings

We summarize the detailed hyperparameter configurations for the model architecture, meta-training phase, and TTA phase in Table S2. All hyperparameters were selected based on preliminary experiments on the validation set.

Details of training and testing

Hardware and Software Environment: All experiments were conducted on a high-performance workstation running Ubuntu 22.04 LTS. The system is equipped with an Intel Core i9-14900KF CPU and dual NVIDIA RTX 5090 (32GB) GPUs. We utilized a single GPU (CUDA 12.8) for model training and inference. The code was implemented in Python 3.8 using PyTorch 2.10.0. Key dependencies include NumPy 2.2.6, Pandas 2.3.3, and Scikit-learn 1.7.2.

Data Preprocessing and Splitting: Raw mRNA sequences were processed into three functional segments: 5' UTR, CDS, and 3' UTR. We applied a fixed-length truncation/padding strategy: 5' UTRs were truncated to the last 500bp, CDS to the first 2000bp, and 3' UTRs to the first 1500bp. Shorter sequences were padded with a special token (N). To evaluate cross-tissue generalization, we employed a Tissue-Specific Split strategy. The dataset was randomly partitioned by tissue identity into training (80%), validation (10%), and testing (10%) sets. This ensures that the tissues in the test set were never seen during the meta-training phase.

Meta-Training Phase: During the meta-training phase, the model was trained on the aggregation of all training tissues (MultiTissueDataset). We used the Adam optimizer with an initial learning rate of $1e^{-3}$. A ReduceLROnPlateau scheduler was employed to halve the learning rate if the average PCC on validation tissues did not improve for 5 consecutive epochs. Training was terminated early if validation performance stagnated for 15 epochs (with a minimum guarantee of 50 epochs).

TTA Protocol: For evaluation on unseen tissues (Validation/Test sets), we strictly followed the TTA protocol. For the standard evaluation setting, 50 labeled samples were selected from each target tissue as the Support Set, and the remaining samples were used as the held-out Query Set. 1. Adaptation: All model parameters (encoder, router, basis kernels) were frozen. A new context embedding vector z_{target} was initialized from the mean of training tissue embeddings. We optimized only z_{target} using the Support Set for 50 steps with a learning rate of 0.01 and a batch size of 16. 2. Inference: The optimized z_{target} was then used to generate dynamic weights for predicting TE on the held-out Query Set.

For the w/o TTA ablation reported in Figure 5 of the main text, gradient-based target-domain context optimization was disabled according to the original ablation configuration. This variant is distinct from the mean-source zero-shot inference control evaluated in the additional matched adaptation-strategy experiments.

Evaluation Metrics

To comprehensively assess the predictive performance of ContiTE, we employed four standard regression metrics. Let y_i denote the ground truth log-TE value and $\hat{y}_i$ denote the predicted value for the i-th sample in a dataset of size N. $\bar{y}$ and $\bar{\hat{y}}$ represent the means of the true and predicted values, respectively.

1. Mean Squared Error (MSE): Measures the average squared difference between estimated values and the actual value. Lower is better.

$$\text{MSE} = \frac{1}{N}\sum_{i=1}^{N}(y_i - \hat{y}_i)^2 \tag{11}$$

2. Coefficient of Determination (R^2): Represents the proportion of variance in the dependent variable that is predictable from the independent variables. Higher is better (max is 1.0).

$$R^2 = 1 - \frac{\sum_{i=1}^{N}(y_i - \hat{y}_i)^2}{\sum_{i=1}^{N}(y_i - \bar{y})^2} \tag{12}$$

3. Pearson Correlation Coefficient (PCC): Measures the linear correlation between predictions and targets. It evaluates the model's ability to capture the overall trend.

$$\text{PCC} = \frac{\sum_{i=1}^{N}(y_i - \bar{y})(\hat{y}_i - \bar{\hat{y}})}{\sqrt{\sum_{i=1}^{N}(y_i - \bar{y})^2}\sqrt{\sum_{i=1}^{N}(\hat{y}_i - \bar{\hat{y}})^2}} \tag{13}$$

4. Spearman's Rank Correlation Coefficient (SCC): A non-parametric measure of rank correlation. It assesses how well the relationship between two variables can be described using a monotonic function, which is crucial for ranking mRNA sequences in drug design.

$$\text{SCC} = 1 - \frac{6\sum_{i=1}^{N}d_i^2}{N(N^2 - 1)} \tag{14}$$

where d_i is the difference between the two ranks of each observation.

Computational Efficiency Analysis

We benchmarked the computational resources required for ContiTE's TTA strategy versus standard Full Fine-Tuning (FT). The experiments were conducted on a single NVIDIA RTX 5090 GPU with a support set of 50 samples updated for 50 steps. As presented in Table S3, although the wall-clock time for adaptation is comparable between the two methods on high-end hardware (due to data loading overheads masking the compute difference for small models), ContiTE offers a decisive advantage in Parameter Efficiency. ContiTE reduces the number of tunable parameters by over 1400× (32 vs. 45,429). This substantial reduction in trainable parameters reduces the risk of catastrophic forgetting and overfitting relative to fine-tuning the entire network on a small target-domain dataset. Furthermore, regarding deployment, ContiTE only requires storing a tiny context vector (0.1 KB) for each new tissue, whereas Fine-Tuning requires saving a separate full model copy, demonstrating ContiTE's superior scalability for large-scale multi-tissue atlases.

Convergence Dynamics of TTA

To analyze the optimization stability during the test-time adaptation phase, we visualized the trajectory of the Mean Squared Error (MSE) loss on the support set over 50 adaptation steps. As shown in Figure S1, the loss trajectory exhibits stochastic oscillations. This behavior is expected and attributable to the small batch size (Batch=16) relative to the support set size ($N = 50$), which introduces noise into the gradient estimation. Despite these oscillations, the model actively explores the parameter manifold and consistently reaches low-loss states (MSE < 0.25) throughout the process. The absence of a diverging trend confirms that the learning rate and optimization schedule are stable, allowing the context embedding to rapidly calibrate to the target domain's distribution within a limited computational budget.

Extended Ablation on Support Set Sensitivity

To provide a more granular perspective on the sensitivity to support set size, we augment the sensitivity analysis by introducing additional curves that ablate individual components of the ContiTE architecture (e.g., w/o W-Space, w/o Codon, w/o Decouple). It should be noted that these specific experiments were initially designed as a rapid scan—utilizing only a subset of test tissues and performing a single replication. Consequently, the absolute Pearson Correlation Coefficient (PCC) values depicted in Figure S2 exhibit deviations from the heavily averaged results reported in the comprehensive evaluations within the main text. Nevertheless, the relative performance trajectories and the consistent performance gaps between the full model and its ablated variants remain strictly consistent with our primary tabular findings, further corroborating the critical contribution and stability of each architectural component across varying sample sizes.

Extended Analysis on TTA Optimization Strategies

While a dynamic learning rate was employed during the meta-training phase, the learning rate was kept fixed in our standard Test-Time Adaptation (TTA) protocol. To further explore optimization dynamics during adaptation, we conducted comparative experiments incorporating alternative strategies: introducing a cosine annealing adaptive learning rate for TTA and evaluating the impact of an increased batch size. We utilized the mouse embryonic stem cell dataset (TE_v6.5_mESC)—an unseen tissue type not involved in the training phase—as the target domain. The convergence trajectories are illustrated in Figure S3.

First, the adaptive learning rate demonstrates notable efficacy. As shown by the green trajectory, it successfully smooths the loss oscillations in the later stages of adaptation without sacrificing the initial convergence speed, ultimately achieving the highest predictive accuracy (PCC = 0.844). However, increasing the batch size reveals inherent limitations under our few-shot setting. When the batch size is expanded to 48 (against a total support set of $N = 50$ samples), the model is restricted to approximately a single gradient update per epoch. This insufficient update frequency impedes efficient manifold calibration and slightly degrades the final accuracy (PCC = 0.839). These findings reaffirm our baseline choice (Batch=16, fixed LR) as a robust trade-off, while highlighting adaptive learning rates as a promising direction for future refinement.

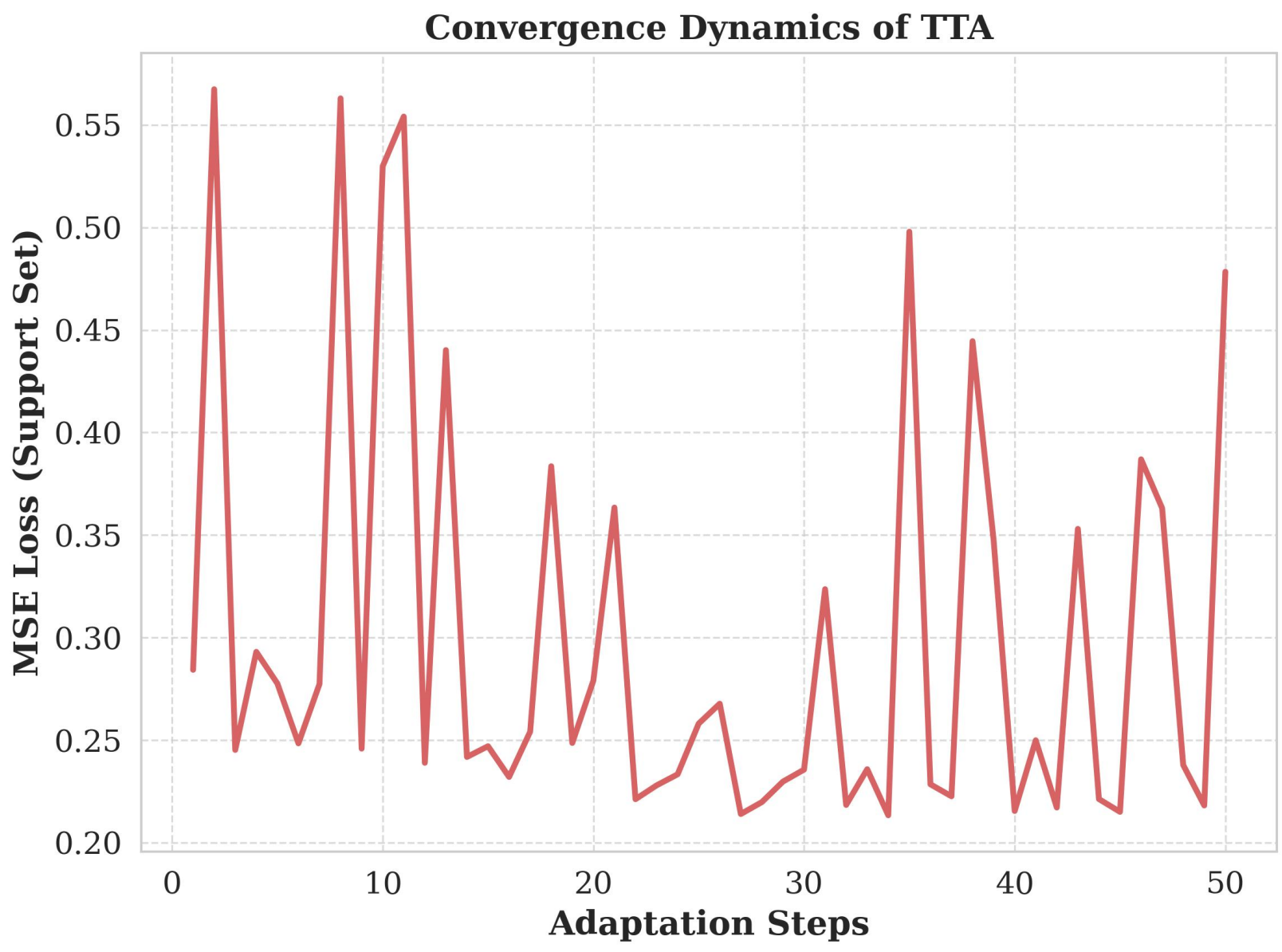

Figure S1 Convergence of context-embedding optimization during Test-Time Adaptation. The curve reports support-set MSE over 50 adaptation steps using a support set of $N = 50$, a batch size of 16, and a learning rate of 0.01. Short-term oscillations reflect stochastic mini-batch updates, whereas the absence of a persistent increasing trend indicates stable optimization under the selected setting.

Table S3 Comparison of computational costs during the adaptation phase.

Method	Tunable Parameters	Storage Overhead	Adaptation Time (50 steps)
Full Fine-Tuning	45,429 (All weights)	∼180 KB / tissue	1.58 s
ContiTE (TTA)	**32 (Context Embed Only)**	**0.1 KB / tissue**	**1.53 s**

Benchmarked on a single NVIDIA RTX 5090 GPU with support set size $N = 50$ and 50 adaptation steps.

Matched Adaptation-Strategy Controls

To separate the effect of target-domain supervision from the choice of adaptation parameterization, we compared five target-domain inference or adaptation strategies using the same pretrained ContiTE checkpoints and identical support/query partitions. The evaluated strategies included mean-source zero-shot inference, regression-head-only tuning, joint context-and-head tuning, full-parameter fine-tuning, and the proposed context-only Test-Time Adaptation (TTA). For every target tissue, $N = 50$ support samples were used, and the experiments were repeated across five random seeds.

Under mean-source zero-shot inference, the target context embedding was initialized as the mean of the source-tissue context embeddings and was directly used for inference without target-domain gradient updates. Context-only TTA updated only the 32-dimensional target context embedding using a learning rate of 1×10^{-2}. Head-only tuning updated only the regression head. For joint context-and-head tuning, the target context embedding and regression head were optimized using learning rates of 1×10^{-2} and 1×10^{-3}, respectively. Full-parameter fine-tuning updated the target context embedding together with all trainable model parameters.

The learning rates for head-only tuning and full fine-tuning were selected exclusively using validation tissues. The selected head-only learning rate was 1×10^{-4} for both species, whereas the full-fine-tuning learning rates were 1×10^{-4} for human and 1×10^{-5} for mouse.

Context-only TTA achieved the highest mean PCC on both the human and mouse atlases, reaching 0.8254 and 0.7355, respectively (Supplementary Table S4). After averaging the five seeds within each tissue and using the 13 target tissues as paired observations, context-only TTA outperformed mean-source zero-shot inference, head-only tuning, joint context-and-head tuning, and full fine-tuning by average PCC values of 0.0327, 0.0236, 0.0424, and 0.0283, respectively. All four comparisons remained significant after Holm correction ($P_{\text{adj}} = 0.000977$).

These controls evaluate alternative adaptation parameterizations within the same pretrained ContiTE architecture. A separate external few-shot comparison in which RiboNN and mRNABERT receive the same target-domain support budget is provided in Supplementary Table S11.

Table S4 Matched target-domain adaptation-strategy controls. Panel A reports mean performance across five random support/query partitions. Panel B reports tissue-level paired Wilcoxon signed-rank tests after averaging the five seeds within each of the 13 target tissues. Multiple comparisons were corrected using the Holm procedure.

Panel A. Predictive performance of matched adaptation strategies

Species	Method	PCC	R^2	SCC	Updated target-domain component
Human	Mean-source zero-shot	0.7962	0.6003	0.7887	None
Human	Head-only	0.8062	0.6440	0.8003	Regression head
Human	Full fine-tuning	0.7991	0.6321	0.7927	All model parameters and target context
Human	Context+Head	0.7935	0.6147	0.7870	Target context and regression head
Human	Context-only	0.8254	0.6714	0.8196	Target context
Mouse	Mean-source zero-shot	0.6987	0.2597	0.6829	None
Mouse	Head-only	0.7066	0.4419	0.6903	Regression head
Mouse	Full fine-tuning	0.7049	0.3548	0.6889	All model parameters and target context
Mouse	Context+Head	0.6809	0.4201	0.6640	Target context and regression head
Mouse	Context-only	0.7355	0.5038	0.7190	Target context

Panel B. Paired comparisons with context-only TTA

Comparison with context-only TTA	PCC difference	Raw P	Holm-adjusted P	No. of tissues
Mean-source zero-shot	0.0327	0.000244	0.000977	13
Head-only	0.0236	0.000244	0.000977	13
Context+Head	0.0424	0.000244	0.000977	13
Full fine-tuning	0.0283	0.000488	0.000977	13

PCC differences are calculated as context-only TTA minus the corresponding control. Statistical tests used the mean of five seeds within each tissue as the paired observation.

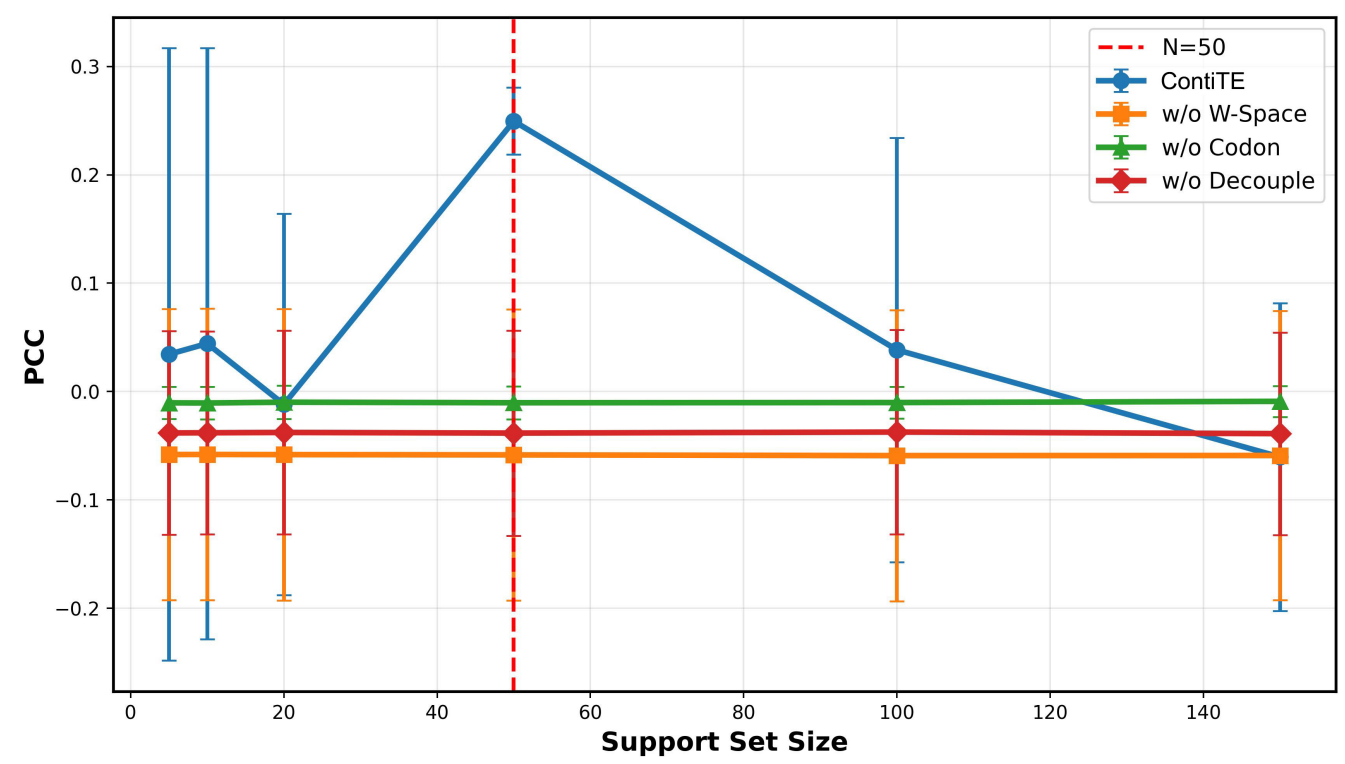

Figure S2 Support-set sensitivity of ContiTE and its ablated variants. PCC is shown for the full ContiTE model and the w/o W-Space, w/o Codon, and w/o Decouple variants across the evaluated support-set sizes. Error bars indicate variability across the subset of target tissues included in this rapid-scan analysis, and the red dashed line marks the standard setting of $N = 50$. This exploratory analysis was conducted using a subset of target tissues and a single replication; therefore, it is intended to compare relative performance trends rather than provide absolute values directly comparable with the averaged results reported in the main text. No statistical significance testing was performed.

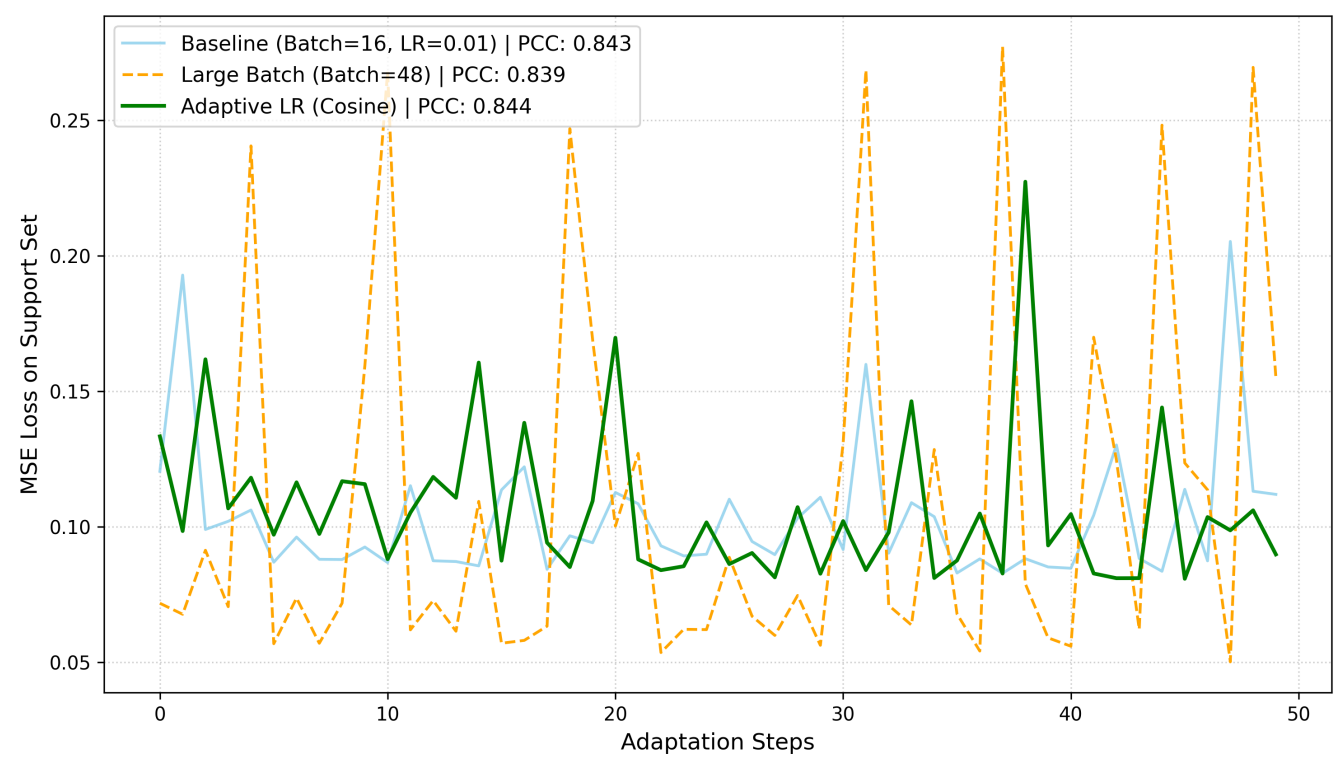

Figure S3 TTA Convergence Dynamics Comparison on the unseen TE_v6.5_mESC dataset. The plot compares the baseline setting (Batch=16, fixed LR) against a large batch setting (Batch=48) and an adaptive learning rate strategy (Cosine Annealing).

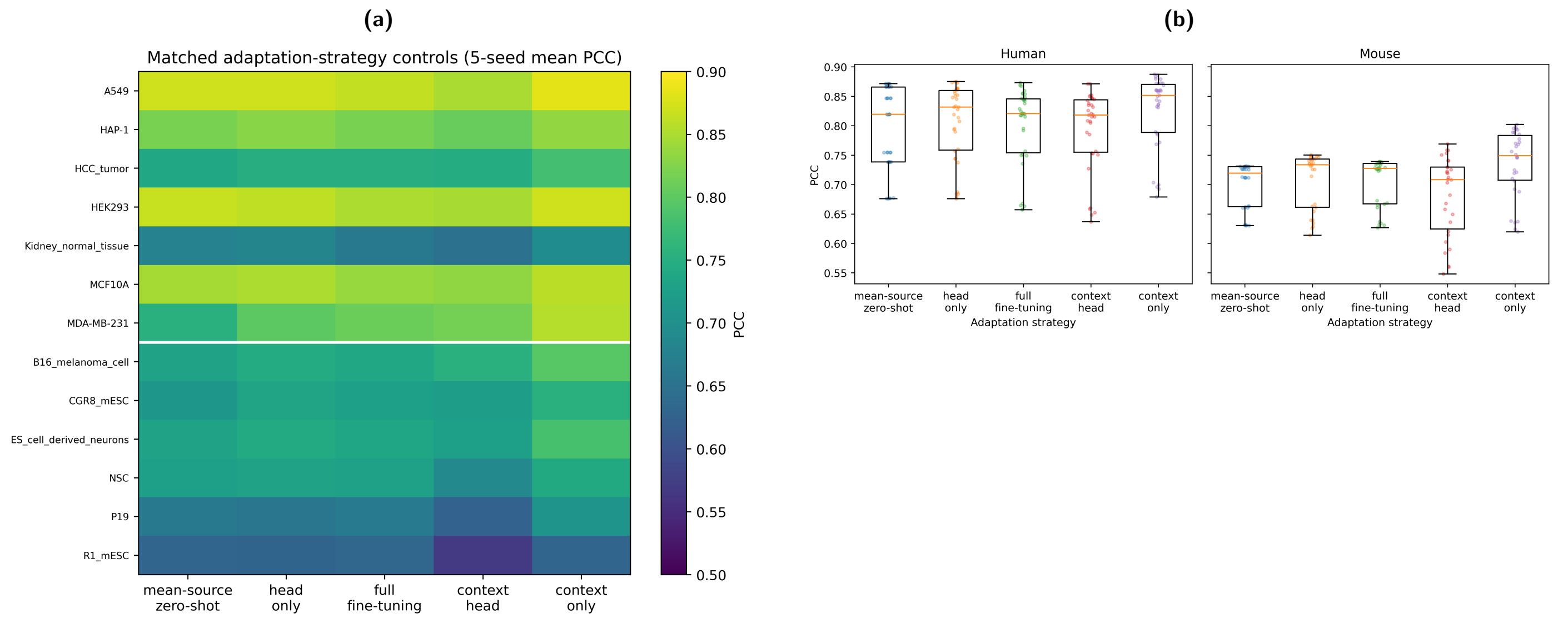

Figure S4 Matched target-domain adaptation-strategy controls. (a) Heatmap of the five-seed mean PCC values for mean-source zero-shot inference, head-only tuning, full fine-tuning, joint context-and-head tuning, and context-only TTA across the 13 target tissues. (b) Distribution of tissue-level PCC values for the five strategies in the human and mouse atlases. All adapted strategies used identical $N = 50$ support/query partitions.

Target-to-Source Manifold Distance and Adaptation Benefit

We investigated whether predictive performance varied with the distance between an unseen target tissue and the source-tissue representations. For each target tissue, we extracted the adapted 32-dimensional context embedding and the corresponding four-dimensional mixing-coefficient vector. The target-to-source distance was defined as the minimum Euclidean distance between the target mixing-coefficient vector and those of all source tissues. Distances were calculated in the original coefficient space rather than in the two-dimensional t-SNE projection, because t-SNE does not preserve global distances.

PCC gain was calculated as the difference between the PCC after $N = 50$ context-only TTA and that obtained by mean-source zero-shot inference. Associations with absolute TTA PCC and PCC gain were evaluated using Spearman rank correlation, two-sided permutation tests, and bootstrap confidence intervals.

Across all 13 target tissues, target-to-source distance was not significantly associated with absolute TTA PCC ($\rho = -0.2473$, permutation $P = 0.4192$). In contrast, distance was positively associated with the PCC gain produced by TTA over mean-source zero-shot inference ($\rho = 0.6758$, permutation $P = 0.0127$, bootstrap 95% CI: 0.2605–0.8592). The associations were not significant when the human and mouse tissues were analyzed separately. The combined result is therefore interpreted as exploratory evidence that context calibration may provide larger benefits for less well-represented target tissues, rather than as a general cross-species law or evidence of monotonic performance decay.

Table S5 Associations between target-to-source mixing-coefficient distance and predictive performance.

Analysis range	Outcome	No. of tissues	Spearman ρ	Permutation P	Bootstrap 95% CI
Human	TTA PCC	7	-0.1429	0.78242	[-1.0000, 0.8824]
Human	PCC gain	7	0.6071	0.16568	[-0.2222, 0.9608]
Mouse	TTA PCC	6	0.3143	0.55814	[-1.0000, 1.0000]
Mouse	PCC gain	6	0.7143	0.14209	[-0.0909, 1.0000]
Overall	TTA PCC	13	-0.2473	0.41916	[-0.7865, 0.4118]
Overall	PCC gain	13	0.6758	0.01270	[0.2605, 0.8592]

PCC gain was defined as PCC after $N = 50$ context-only TTA minus PCC under mean-source zero-shot inference. Permutation tests were two-sided.

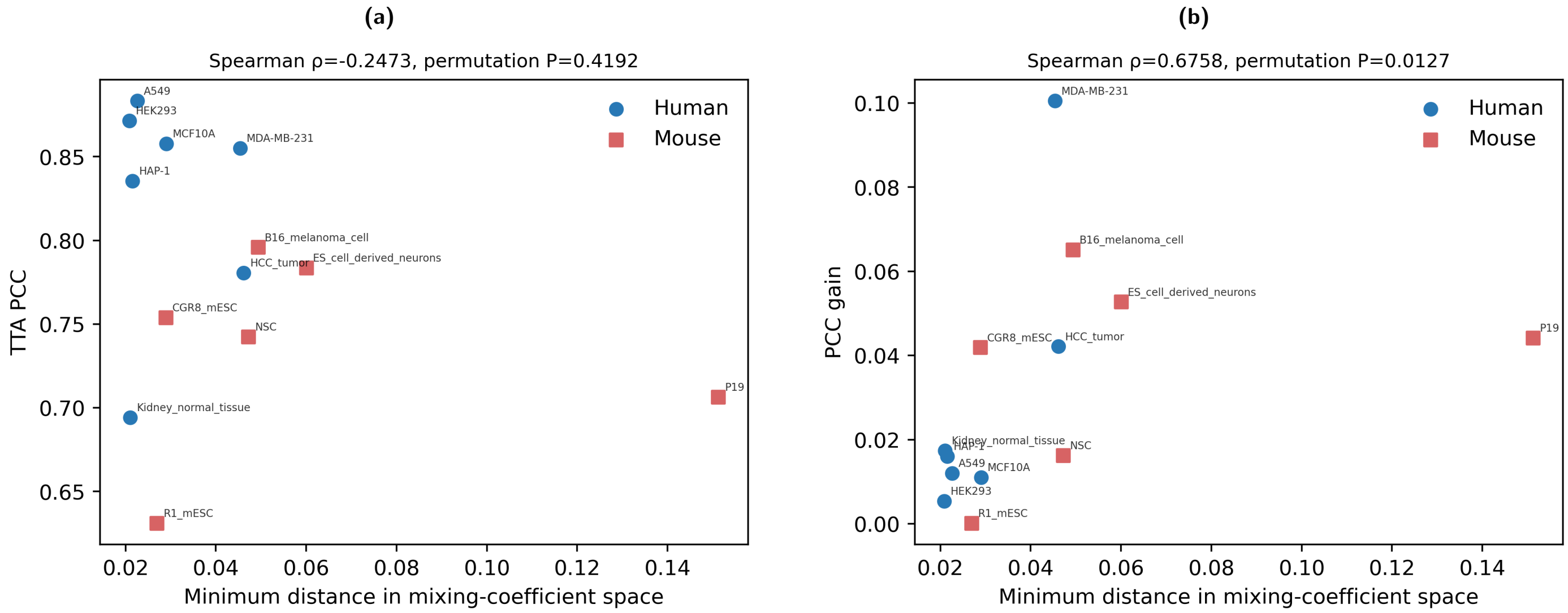

Figure S5 Relationship between target-to-source manifold distance and predictive performance. (a) Minimum target-to-source distance in the original mixing-coefficient space versus PCC after $N = 50$ context-only TTA. (b) The same distance versus the PCC gain over mean-source zero-shot inference. Each point represents one target tissue. Human and mouse tissues are indicated using different symbols. Reported P values were obtained from two-sided permutation tests.

Robustness to Context-Embedding Initialization

We compared source-mean, zero-vector, random Gaussian, randomly selected source-tissue, and support-aware best-source initialization. All other TTA settings were fixed, and only the 32-dimensional target context embedding was optimized for 50 steps using $N = 50$ support samples.

Source-mean initialization achieved average PCC values of 0.8254 and 0.7355 in the human and mouse atlases, respectively. Zero-vector and support-aware best-source initialization did not differ significantly from source-mean initialization. In contrast, random Gaussian and random-source initialization reduced average PCC by 0.0149 and 0.0143, respectively, with Holm-adjusted P values of 0.000977 and 0.005127. These results indicate that ContiTE is robust to deterministic initialization strategies, whereas poorly matched random initialization can produce a small but detectable performance loss.

Table S6 Robustness of ContiTE to target-context initialization. Panel A summarizes predictive performance. Panel B reports paired comparisons with source-mean initialization.

Panel A. Predictive performance under different initialization strategies

Species	Initialization	Mean PCC	SD	Mean R^2	n
Human	Best source	0.8271	0.0607	0.6788	35
Human	Source mean	0.8254	0.0630	0.6714	35
Human	Zero vector	0.8223	0.0668	0.6708	35
Human	Random Gaussian	0.8082	0.0733	0.6461	35
Human	Random source	0.8072	0.0733	0.6407	35
Mouse	Source mean	0.7355	0.0573	0.5038	30
Mouse	Zero vector	0.7347	0.0586	0.5032	30
Mouse	Best source	0.7311	0.0607	0.5026	30
Mouse	Random source	0.7258	0.0655	0.4445	30
Mouse	Random Gaussian	0.7233	0.0752	0.4795	30

Panel B. Paired comparisons with source-mean initialization

Initialization	Mean PCC difference	Raw P	Holm-adjusted P	Interpretation
Best source	-0.0011	0.497314	0.994629	Not significant
Random Gaussian	-0.0149	0.000244	0.000977	Significant reduction
Random source	-0.0143	0.001709	0.005127	Significant reduction
Zero vector	-0.0020	0.684814	0.994629	Not significant

In Panel B, PCC differences are calculated as the alternative initialization minus source-mean initialization.

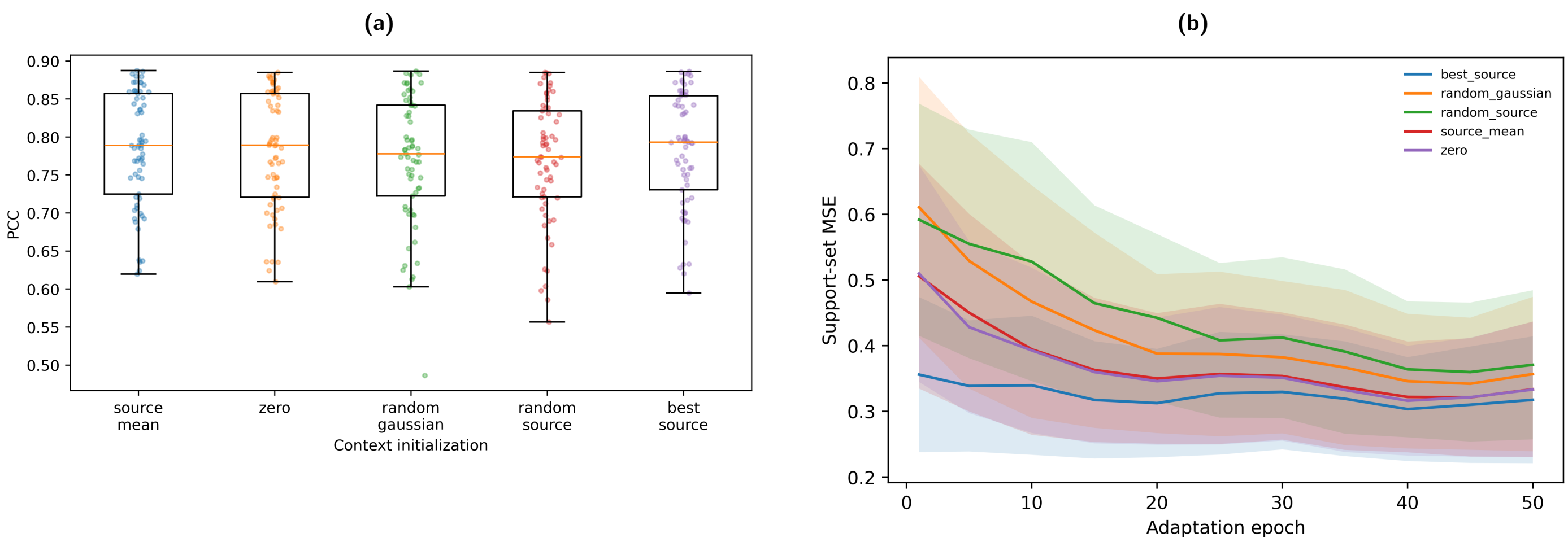

Figure S6 Robustness to target-context initialization. (a) Distribution of final PCC values under source-mean, zero-vector, random Gaussian, random-source, and support-aware best-source initialization. (b) Support-set MSE trajectories over 50 adaptation steps. Curves summarize results across target tissues and random seeds.

Robustness to Support-Set Noise and Tissue Heterogeneity

We conducted computational stress tests by perturbing only the target-domain support set while leaving the query set unchanged. Label noise was simulated by adding Gaussian perturbations proportional to the support-label standard deviation at relative levels of 0.1, 0.2, and 0.3. Tissue heterogeneity was simulated by replacing 10%, 20%, or 30% of the target support samples with samples from other tissues of the same species.

Support-label noise caused only minimal changes in average PCC, with maximum reductions of 0.0023 and 0.0010 in the human and mouse atlases, respectively; none of these differences were statistically significant. Tissue contamination caused modest performance degradation. In the human atlas, 20% and 30% contamination reduced average PCC by 0.0087 and 0.0109, respectively, with Holm-adjusted $P = 0.046875$. No contamination level reached significance in the mouse atlas. These experiments assess computational robustness to label perturbation and tissue mixture and should not be interpreted as direct simulations of sequencing depth, clinical heterogeneity, or batch effects.

Table S7 Computational robustness to support-label noise and cross-tissue contamination. Panel A reports predictive performance, and Panel B reports paired comparisons with the corresponding unperturbed condition.

Panel A. Predictive performance under support-set perturbations

Species	Perturbation	Level	Mean PCC	PCC reduction from clean	Interpretation
Human	Label noise	0.0	0.8254	0.0000	Clean
Human	Label noise	0.1	0.8247	0.0007	Minimal change
Human	Label noise	0.2	0.8256	-0.0002	Minimal change
Human	Label noise	0.3	0.8231	0.0023	Minimal change
Mouse	Label noise	0.0	0.7355	0.0000	Clean
Mouse	Label noise	0.1	0.7349	0.0005	Minimal change
Mouse	Label noise	0.2	0.7347	0.0007	Minimal change
Mouse	Label noise	0.3	0.7344	0.0010	Minimal change
Human	Tissue contamination	0.0	0.8254	0.0000	Clean
Human	Tissue contamination	0.1	0.8229	0.0025	Modest reduction
Human	Tissue contamination	0.2	0.8167	0.0087	Modest reduction
Human	Tissue contamination	0.3	0.8145	0.0109	Modest reduction
Mouse	Tissue contamination	0.0	0.7355	0.0000	Clean
Mouse	Tissue contamination	0.1	0.7328	0.0027	Modest reduction
Mouse	Tissue contamination	0.2	0.7346	0.0009	Minimal change
Mouse	Tissue contamination	0.3	0.7287	0.0067	Modest reduction

Panel B. Paired comparisons with the clean condition

Species	Perturbation	Level	PCC difference	Holm-adjusted P	Interpretation
Human	Label noise	0.1	-0.0007	0.937500	Not significant
Human	Label noise	0.2	0.0002	0.937500	Not significant
Human	Label noise	0.3	-0.0023	0.656250	Not significant
Mouse	Label noise	0.1	-0.0005	1.000000	Not significant
Mouse	Label noise	0.2	-0.0007	1.000000	Not significant
Mouse	Label noise	0.3	-0.0010	1.000000	Not significant
Human	Tissue contamination	0.1	-0.0025	0.109375	Not significant
Human	Tissue contamination	0.2	-0.0087	0.046875	Significant reduction
Human	Tissue contamination	0.3	-0.0109	0.046875	Significant reduction
Mouse	Tissue contamination	0.1	-0.0027	0.937500	Not significant
Mouse	Tissue contamination	0.2	-0.0009	0.937500	Not significant
Mouse	Tissue contamination	0.3	-0.0067	0.937500	Not significant

Label-noise levels are relative to the support-label standard deviation. Tissue-contamination levels denote the proportion of support samples replaced with samples from other tissues of the same species. Query sets remained unchanged and unperturbed.

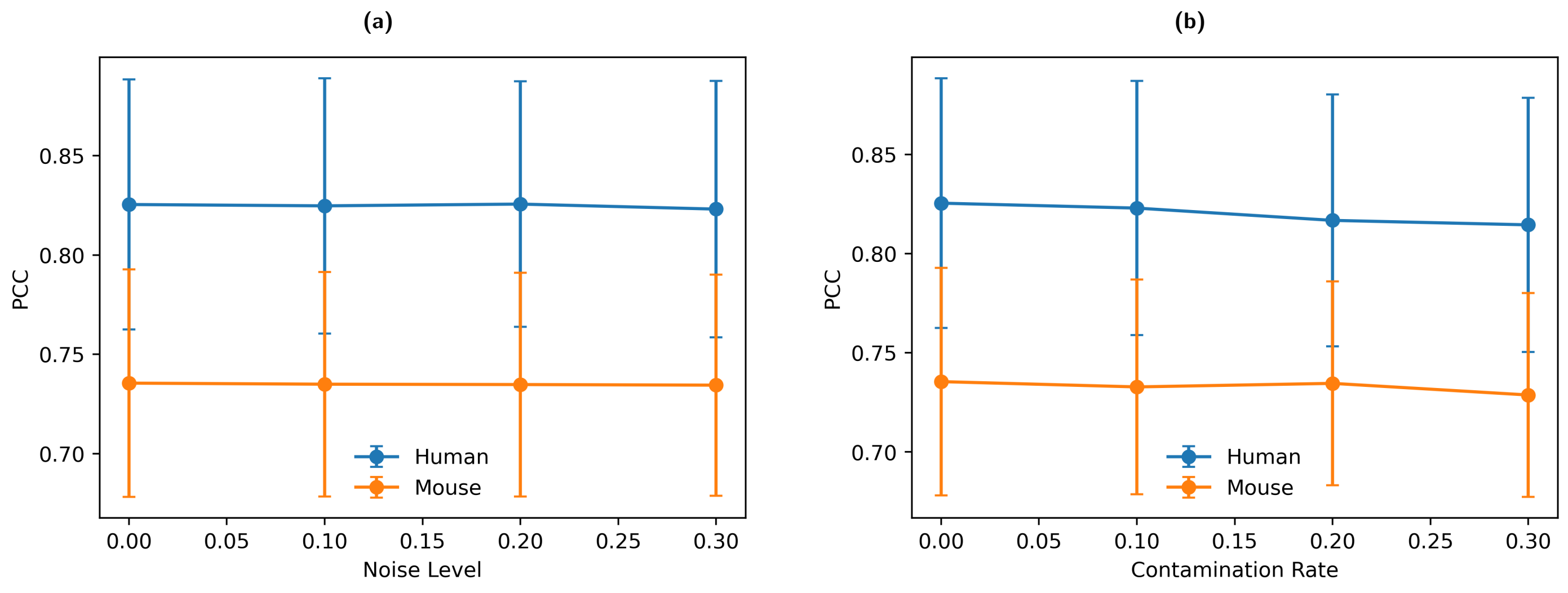

Figure S7 Computational robustness to support-set perturbations. (a) Average PCC under increasing levels of Gaussian support-label noise. (b) Average PCC when increasing proportions of the target support set are replaced with samples from other tissues of the same species. Query sets remained unchanged and unperturbed. Error bars summarize variability across target tissues and random seeds.

Sensitivity to the Number of Basis Kernels

We performed a single-run computational sensitivity analysis on the human atlas using $K = 2$, 4, and 8 basis kernels. The $K = 2$ and $K = 8$ models were newly trained for up to 50 epochs, whereas $K = 4$ corresponded to the checkpoint used in the main experiments. Except for the number of basis kernels, the tissue split, preprocessing procedure, context dimensionality, optimization settings, batch size, and loss function were kept unchanged. Validation tissues were used for checkpoint selection, and test tissues were not used during model selection.

The validation PCC values were 0.6980, 0.7627, and 0.7471 for $K = 2$, 4, and 8, respectively, and the corresponding test PCC values were 0.7950, 0.8254, and 0.8138. Thus, $K = 4$ provided the highest validation and test performance under the evaluated protocol. The lower performance of $K = 2$ suggests reduced representational capacity, whereas increasing the basis number to eight did not provide further improvement. Because this analysis was restricted to the human atlas and a single training run, it provides computational sensitivity evidence rather than a multi-seed or cross-species hyperparameter benchmark.

Table S8 Sensitivity to the number of atomic basis kernels on the human atlas.

K	Best epoch	Val PCC	Val R^2	Test PCC	Test R^2	Training time (min)	Peak memory (MB)
2	40	0.6980	0.3091	0.7950	0.6015	31.9	172.8
4	–	0.7627	0.5835	0.8254	0.6714	–	–
8	50	0.7471	0.5425	0.8138	0.6430	33.2	173.6

The $K = 2$ and $K = 8$ results were obtained from single newly trained models. The $K = 4$ result corresponds to the checkpoint used in the main experiments; its original training-time and peak-memory measurements were not recomputed.

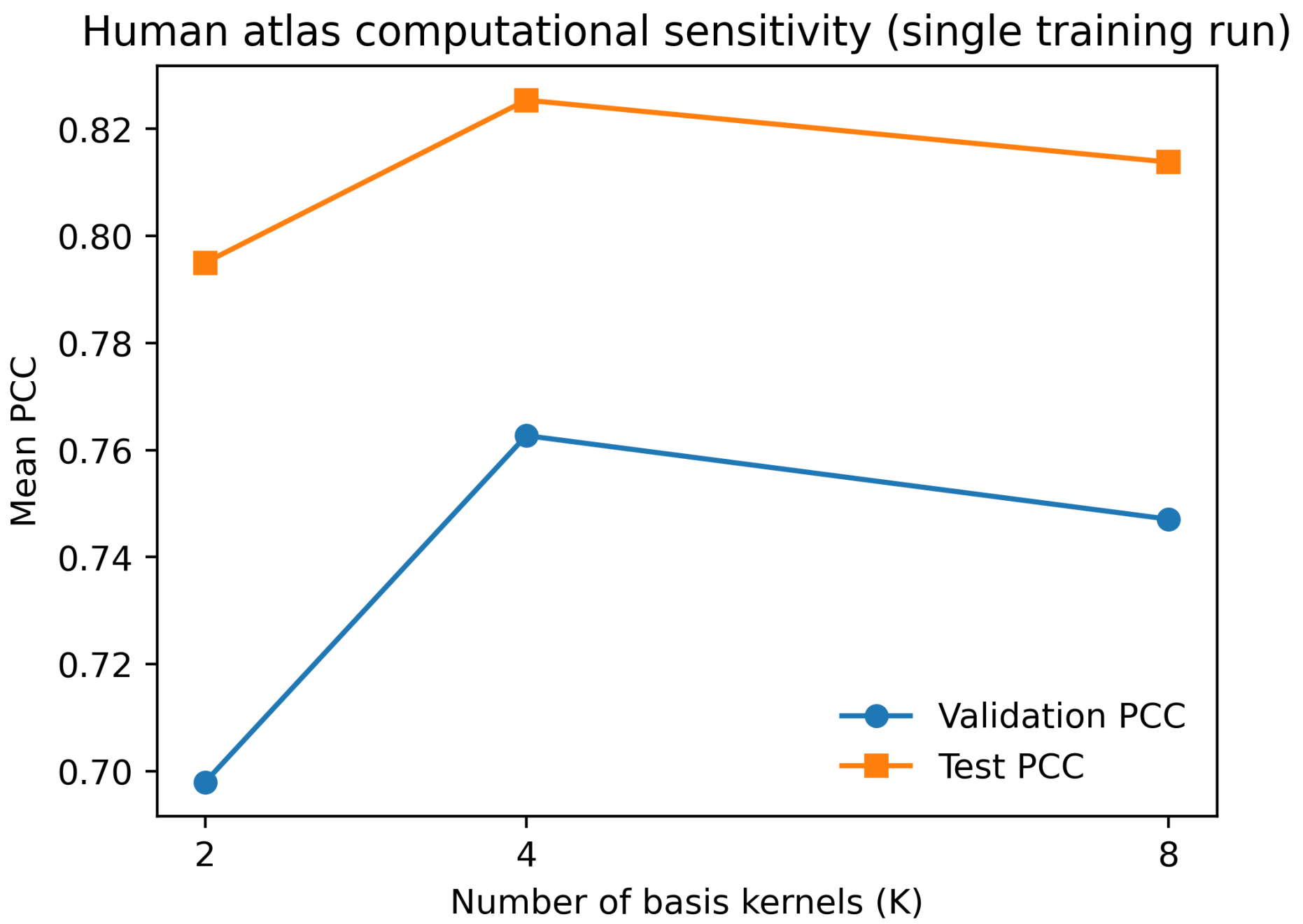

Figure S8 Sensitivity to the number of atomic basis kernels on the human atlas. Validation and test PCC values are shown for $K = 2, 4$, and 8. The $K = 2$ and $K = 8$ results were obtained from single training runs, whereas $K = 4$ corresponds to the checkpoint used in the main experiments.

Mean-Source Zero-Shot and Extremely Low-Sample Adaptation

To assess adaptation when target-domain labels are extremely limited, we evaluated mean-source zero-shot inference and support-set sizes of $N = 1, 5, 20$, and 50. Under mean-source zero-shot inference, the target context was initialized as the mean of the source-tissue context embeddings and used directly without target-domain gradient updates. This control is distinct from the original w/o TTA ablation reported in Figure 5 of the main text.

For $N \geq 1$, the support sets were nested within the same deterministic sample ordering. The zero-shot result was evaluated using the query set corresponding to the $N = 50$ condition. Human average PCC changed from 0.7962 under mean-source zero-shot inference to 0.7802, 0.7868, 0.8143, and 0.8254 at $N = 1, 5, 20$, and 50, respectively. Mouse average PCC changed from 0.6987 to 0.6954, 0.7163, 0.7314, and 0.7355. Single-sample adaptation was less stable than mean-source zero-shot inference, whereas performance gains became more consistent at $N = 20$ and $N = 50$. Because each support size produces a different complementary query set, the curve is interpreted as a sample-efficiency trend rather than as a strictly paired comparison of adjacent points.

Table S9 Mean-source zero-shot inference and extremely low-sample adaptation.

Species	Support size (N)	Mean PCC	SD	Mean R^2	n
Human	0	0.7962	0.0696	0.6003	35
Human	1	0.7802	0.0886	0.5462	35
Human	5	0.7868	0.0839	0.5751	35
Human	20	0.8143	0.0704	0.6500	35
Human	50	0.8254	0.0630	0.6714	35
Mouse	0	0.6987	0.0392	0.2597	30
Mouse	1	0.6954	0.0590	0.2617	30
Mouse	5	0.7163	0.0610	0.3902	30
Mouse	20	0.7314	0.0521	0.4819	30
Mouse	50	0.7355	0.0573	0.5038	30

$N = 0$ denotes mean-source zero-shot inference. For $N \geq 1$, the target context embedding was optimized using context-only TTA. Different support sizes produced different complementary query sets.

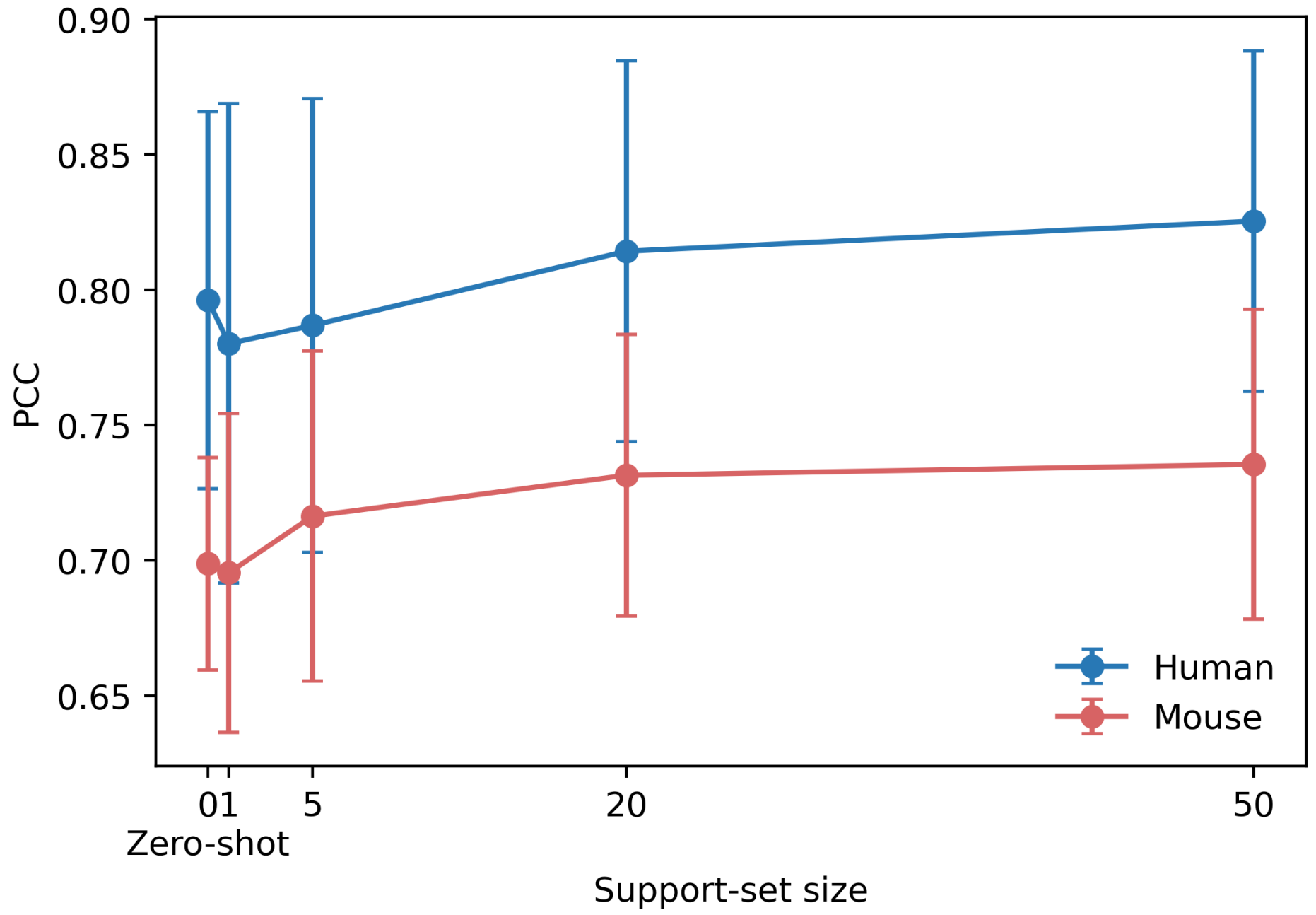

Figure S9 Mean-source zero-shot inference and extremely low-sample target-domain adaptation. Average PCC values are shown for mean-source zero-shot inference ($N = 0$) and context-only TTA using $N = 1$, 5, 20, and 50 support samples. Error bars summarize variability across target tissues and random seeds. Because the query set changes with the support size, the curves represent overall data-efficiency trends rather than strictly paired comparisons between adjacent points.

Empirical Diversity and Redundancy of the Basis Kernels

To empirically examine the learned basis set without assuming strict orthogonality, we extracted the four basis kernels from the 5' UTR, CDS, and 3' UTR dynamic convolution branches and calculated pairwise cosine-similarity Gram matrices.

The CDS branch exhibited the lowest mean off-diagonal similarity in both species (0.2425 for human and 0.3598 for mouse), indicating comparatively greater basis diversity. The UTR branches showed higher similarity, particularly the mouse 5' UTR branch, for which the mean and maximum off-diagonal similarities reached 0.7878 and 0.9362, respectively. These results indicate that the effective diversity of the basis kernels varies across sequence branches and that some redundancy is present. The analysis does not support or require strict basis orthogonality.

Table S10 Off-diagonal cosine similarity among learned atomic basis kernels.

Species	Dynamic convolution branch	Mean off-diagonal similarity	Minimum	Maximum
Human	CDS	0.2425	-0.2243	0.5645
Human	3' UTR	0.5486	0.3384	0.6968
Human	5' UTR	0.6665	0.4744	0.8056
Mouse	CDS	0.3598	0.1120	0.5339
Mouse	3' UTR	0.6702	0.5376	0.8484
Mouse	5' UTR	0.7878	0.6515	0.9362

Similarities were calculated after flattening the four basis kernels within each dynamic convolution branch. Lower off-diagonal similarity indicates greater empirical diversity.

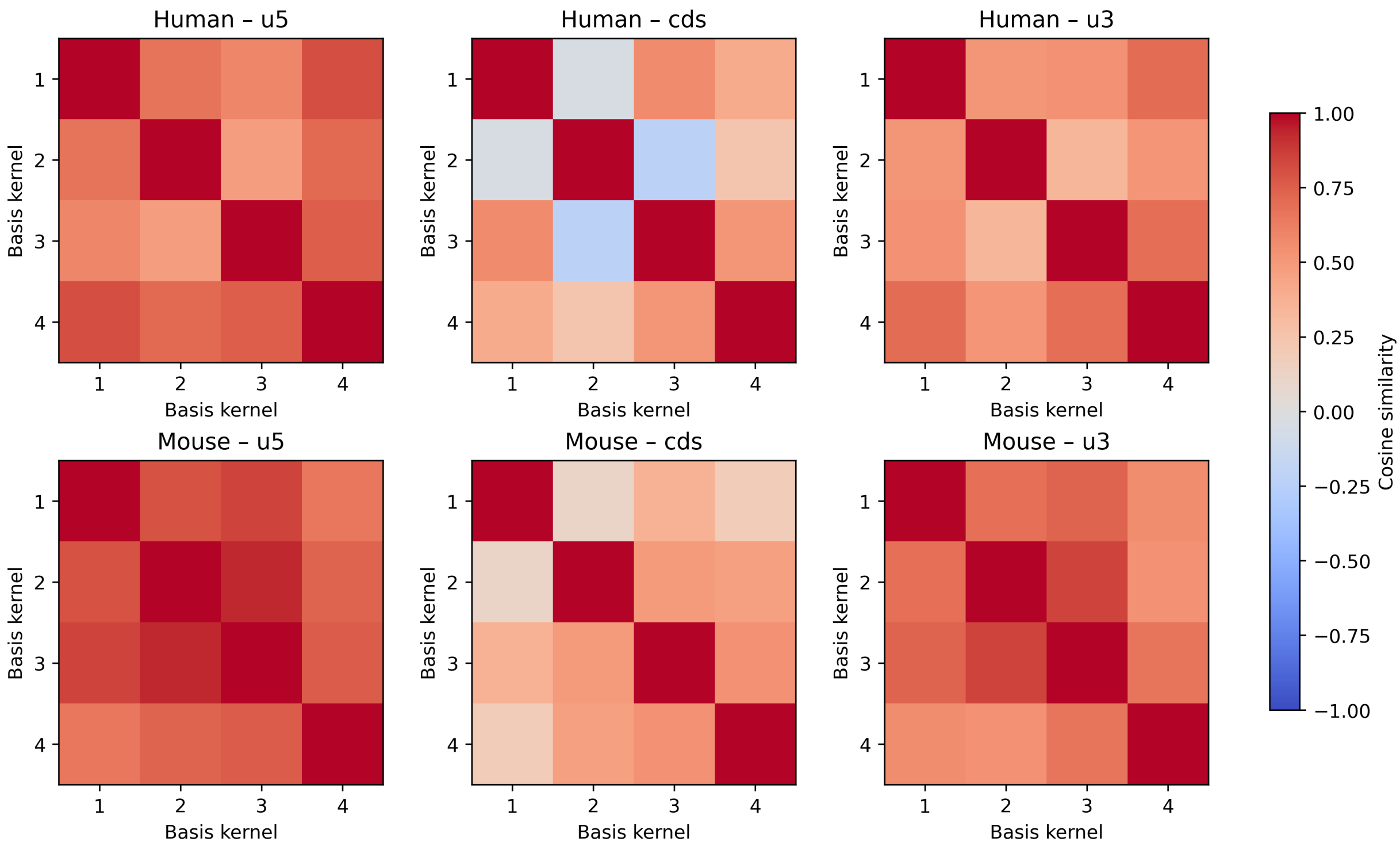

Figure S10 Cosine-similarity Gram matrices of the learned atomic basis kernels. Pairwise similarities are shown for the 5' UTR, CDS, and 3' UTR dynamic convolution branches in the human and mouse models. Lower off-diagonal similarity indicates greater empirical diversity among the learned basis kernels.

Matched Few-Shot Adaptation of External Baselines

To provide external baselines with the same target-domain adaptation opportunity as ContiTE, we additionally evaluated RiboNN and mRNABERT using output-head tuning. Each baseline was trained using only the source tissues employed in the ContiTE meta-training split. The validation tissues were used for checkpoint and adaptation learning-rate selection, whereas the test tissues remained completely unseen during source-domain training and model selection.

For each unseen target tissue, the sequence encoder of the external baseline was frozen and only the final regression head was optimized. RiboNN, mRNABERT, and ContiTE used identical $N = 50$ support samples and the same held-out query samples for every tissue and random seed. All adaptation procedures used 50 optimization steps. The output-head learning rate was selected from $\{1 \times 10^{-4}, 1 \times 10^{-3}, 1 \times 10^{-2}\}$ based exclusively on the validation tissues. The experiments were repeated using the same five random seeds as the matched adaptation-strategy controls.

Supplementary Table S11 summarizes the performance under matched target-domain supervision.

Table S11 Few-shot adaptation comparison under matched target-domain supervision. Panel A reports performance averaged across the target tissues and random seeds. Panel B reports tissue-level paired comparisons with ContiTE after averaging the random seeds within each of the 13 target tissues. Multiple comparisons are corrected using the Holm procedure.

Panel A. Performance under matched few-shot adaptation

Species	Method	Target-domain adaptation	MSE	R^2	PCC	SCC
Human	RiboNN	Output-head tuning	0.9100	0.3820	0.6900	0.6760
Human	mRNABERT	Output-head tuning	0.7500	0.5030	0.7420	0.7300
Human	ContiTE	Context-only TTA	0.5600	0.6714	0.8254	0.8196
Mouse	RiboNN	Output-head tuning	1.1400	0.2180	0.6120	0.5960
Mouse	mRNABERT	Output-head tuning	0.9700	0.3410	0.6630	0.6480
Mouse	ContiTE	Context-only TTA	0.7800	0.5038	0.7355	0.7190
Overall	RiboNN	Output-head tuning	1.0162	0.3063	0.6540	0.6391
Overall	mRNABERT	Output-head tuning	0.8515	0.4282	0.7055	0.6922
Overall	ContiTE	Context-only TTA	0.6615	0.5941	0.7839	0.7732

Panel B. Paired PCC comparisons with ContiTE

Comparison	Mean PCC difference	Raw P	Holm-adjusted P	No. of tissues
ContiTE vs. RiboNN	0.1299	0.000244	0.000488	13
ContiTE vs. mRNABERT	0.0784	0.000488	0.000488	13

All methods used identical $N = 50$ target-domain support samples and the same held-out query samples for each tissue and random seed. RiboNN and mRNABERT were adapted by updating only their regression heads, whereas ContiTE updated only the 32-dimensional target context embedding.

Human and mouse summaries are unweighted averages across the corresponding target tissues. The overall values are averages across all seven human and six mouse target tissues.

In Panel B, PCC differences are calculated as ContiTE minus the corresponding external baseline. Statistical testing uses each target tissue as one paired observation after averaging across random seeds.
